# Polycystin‐1 Mutant Alters Mechanotransduction in Response to Collagen and Extracellular Matrix Stiffness via Daam1‐Dependent Microfilament Remodeling

**DOI:** 10.1002/advs.202509846

**Published:** 2025-08-11

**Authors:** Jiaofeng Zhou, Yibing Deng, Jie Mei, Mengyun Wan, Ningyi Xue, Xinyu Zong, Ji Zhou, Junli Ding, Ting Yan, Yu Jiang, Tiansong Xia, Peng Zheng, Yichao Zhu

**Affiliations:** ^1^ Department of Physiology School of Basic Medical Sciences Nanjing Medical University Nanjing Jiangsu 211166 China; ^2^ State Key Laboratory of Coordination Chemistry School of Chemistry and Chemical Engineering Chemistry and Biomedicine Innovation Centre (ChemBIC) Nanjing University Nanjing Jiangsu 210023 China; ^3^ The First Clinical Medicine College Nanjing Medical University Nanjing Jiangsu 211166 China; ^4^ Department of Breast Surgery The First Affiliated Hospital of Nanjing Medical University Nanjing Jiangsu 210029 China; ^5^ Pancreas Center The First Affiliated Hospital of Nanjing Medical University Nanjing Jiangsu 210029 China; ^6^ Department of Oncology The Affiliated Wuxi People's Hospital of Nanjing Medical University Wuxi Medical Center Nanjing Medical University Wuxi Jiangsu 214023 China; ^7^ Safety Assessment and Research Center for Drug Pesticide and Veterinary Drug of Jiangsu Province Nanjing Medical University Nanjing Jiangsu 211166 China; ^8^ Department of Pharmacology and Chemical Biology University of Pittsburgh School of Medicine Pittsburgh Pennsylvania 15261 USA; ^9^ Department of General Surgery The Affiliated Taizhou People's Hospital of Nanjing Medical University Taizhou School of Clinical Medicine Nanjing Medical University Taizhou Jiangsu 225300 China

**Keywords:** Daam1, extracellular matrix, mechanotransduction, microfilament, polycystin‐1

## Abstract

Extracellular matrix (ECM) stiffness‐mediated mechanotransduction is a common signaling scheme in both physiological and pathological contexts; however, its molecular mechanisms remain incompletely understood. Polycystin‐1 is a transmembrane protein that is known to participate in mechano‐transduction. Here, it is demonstrated that, in response to extracellular collagen and increased ECM stiffness, polycystin‐1 interacts with disheveled‐associated activator of morphogenesis 1 (Daam1), a cytoskeletal regulator, thereby promoting microfilament remodeling, cellular protrusion formation, and enhanced motility of tumor cells through activating the RhoA signaling axis. Wild‐type polycystin‐1 is susceptible to proteolytic cleavage at the G protein‐coupled receptor proteolysis site. Using atomic force microscopy‐based single‐molecule force spectroscopy, direct evidence is provided that polycystin‐1 variants R3039H and L3048H exhibit reduced cleavage susceptibility in vitro. Notably, R3039H is associated with lymphatic and distant metastasis in breast cancer and augments mechanotransduction by facilitating the nuclear translocation of Yes‐associated protein and upregulating the expression of connective tissue growth factor and collagen in tumor cells and cancer‐associated fibroblasts, respectively. Collectively, our findings identify polycystin‐1 as a mechanosensor of collagen and ECM stiffness that modulates tumor cell migration via the Daam1/RhoA/YAP signaling cascade.

## Graphical abstract

Polycystin‐1 (PC1) senses collagen and ECM stiffness to promote tumor migration via Daam1‐mediated actin remodeling. A cleavage‐resistant PC1 mutant enhances YAP nuclear translocation and CTGF expression, which activates CAFs and further stiffens the ECM, forming a feed‐forward loop that drives tumor progression and metastasis.

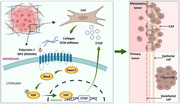

## 1 Introduction

Cellular mechanotransduction is the process by which cells sense and convert mechanical cues such as extracellular matrix (ECM) stiffness, hydrostatic pressure, tensile and shear forces, and fluid viscosity into biochemical signals, playing a critical role in numerous physiological and pathological processes.^[^ ^1^ ^]^ Among these cues, ECM stiffness has emerged as a hallmark of the tumor microenvironment, contributing to the malignant transformation and enhanced invasive and metastatic progression of tumor cells.^[^ ^2^ ^]^ Increased ECM stiffness facilitates the activation of specific intracellular signaling pathways that augment cellular motility and promote metastatic dissemination.^[^ ^2^, ^3^, ^4^, ^5^ ^]^ However, the precise molecular mechanisms by which ECM stiffness triggers mechanotransductive signaling remain unclear.

Polycystin‐1, the largest known transmembrane protein in the human proteome, is encoded by the polycystic kidney disease 1 (*PKD1*) gene, which lies within a duplicated region on chromosome 16 and produces an mRNA transcript with a length of ≈14 kb.^[^ ^6^ ^]^ Polycystin‐1 is a mechanosensitive protein that responds to extracellular mechanical cues and mediates cystogenesis in polycystic kidney disease (PKD).^[^ ^7^ ^]^ Functionally, polycystin‐1 forms a heteromeric complex with polycystin‐2, a calcium‐permeable channel localized in the plasma membrane and the endoplasmic reticulum, to mediate calcium‐dependent mechanotransduction.^[^ ^8^ ^]^ However, whether polycystin‐1 senses ECM stiffness and mediates mechanotransduction during tumor progression remains largely unexplored. Polycystin‐1 contains a leucine‐rich repeat (LRR) domain within its extracellular region, which confers collagen‐binding capacity and facilitates cell‐matrix and cell‐cell interactions.^[^ ^9^ ^]^ In this study, we used atomic force microscopy‐based single‐molecule force spectroscopy (AFM‐SMFS) and cellular and biochemical approaches to investigate the mechanotransductive role of polycystin‐1 in tumor biology.

Collagen is the most abundant structural protein in the ECM and interacts with numerous membrane receptors to regulate cell‐matrix signaling.^[^ ^10^, ^11^, ^12^ ^]^ We have previously shown that collagen activates disheveled‐associated activator of morphogenesis 1 (Daam1), a member of the formin protein family, to enhance microfilament dynamics and cell motility.^[^ ^13^ ^]^ However, the upstream molecular mechanisms by which extracellular collagen cues are transmitted to intracellular Daam1 remain to be elucidated. In this study, we demonstrate that polycystin‐1 interacts with Daam1 and acts as a molecular transducer of collagen and ECM stiffness‐derived mechanosignals. This interaction leads to cytoskeletal remodeling, protrusion formation, and enhanced motility of tumor cells, demonstrating a novel polycystin‐1/Daam1‐dependent mechanotransduction axis.

## 2 Results

### 2.1 Polycystin‐1 Modulates Collagen‐Induced Microfilament Remodeling and Tumor Cell Motility

Polycystin‐1 contains an LRR domain within its N‐terminal extracellular region that can bind collagen^[^ ^9^ ^]^ (Figure S1a, Supporting Information). Thus, we hypothesized that polycystin‐1 may respond to collagen stimulation and influence cell behavior. Consistent with this finding, collagen I was abundantly expressed in breast cancer tissues (Figure S1b, Supporting Information). To identify which proteins are bound by polycystin‐1 in response to collagen, HEK‐293T cells were seeded on collagen I‐coated plates, followed by immunoprecipitation‐mass spectrometry of polycystin‐1‐associated proteins. A total of 176 peptides were identified, including several cytoskeletal components, such as myosin‐8, myosin‐9, cytoskeleton‐associated protein 4, and actin (data not shown), suggesting a potential role for polycystin‐1 in microfilament remodeling.

We manipulated polycystin‐1 expression by overexpressing full‐length polycystin‐1 or siRNA‐mediated knockdown (Figure S1c, Supporting Information). To investigate the role of polycystin‐1 in collagen‐mediated cytoskeletal remodeling and cell motility, MCF‐7 and MDA‐MB‐231 breast cancer cells were exposed to increasing concentrations of collagen I. Cell migration and invasion were significantly enhanced at 15 µg cm^−^^2^ collagen compared to 5 µg cm^−^^2^; therefore, these concentrations were used in subsequent experiments (Figure S1d, Supporting Information).

**Figure 1:**
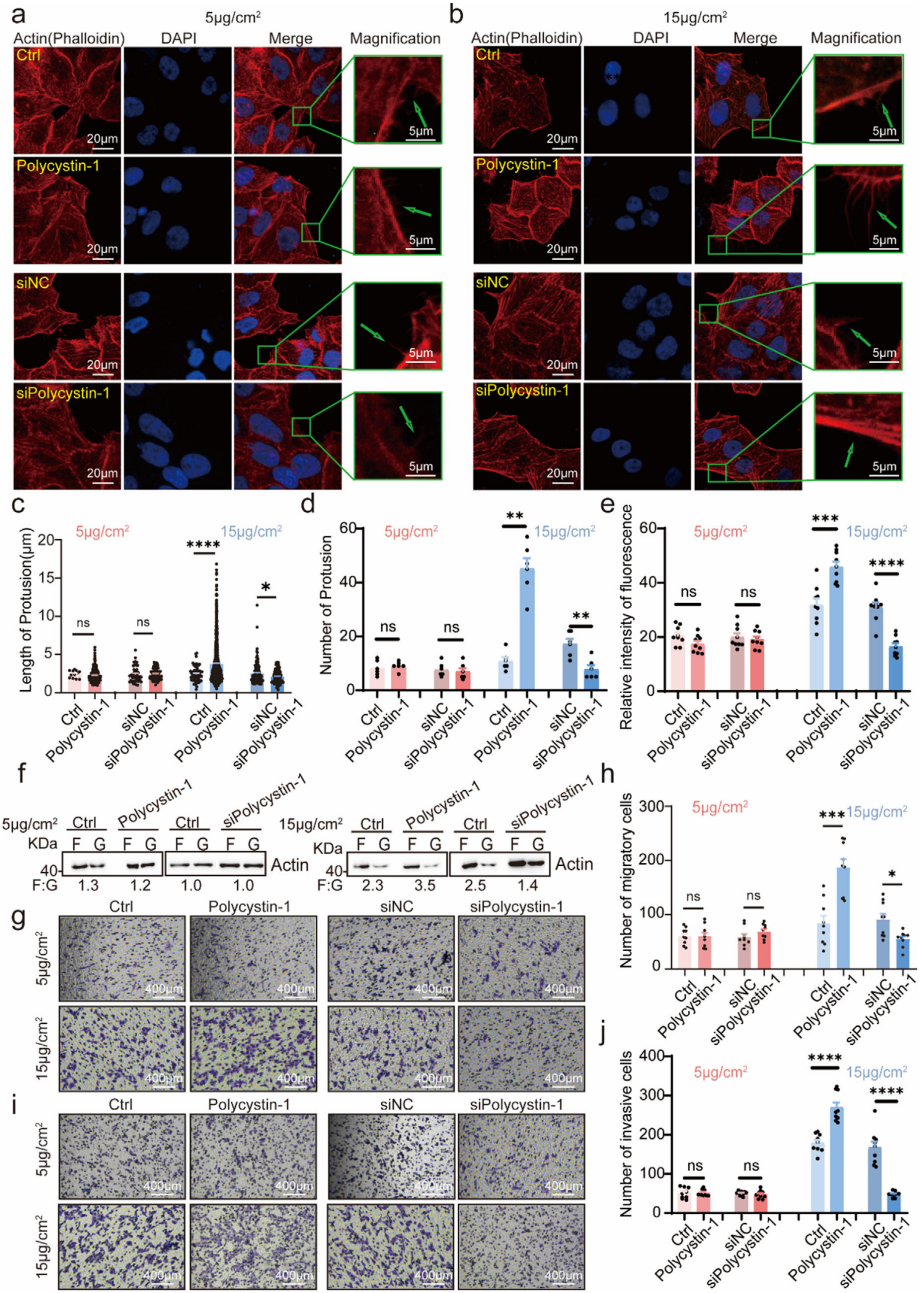
Polycystin‐1 modulates collagen‐induced tumor cell migration and invasion. a–e) Polycystin‐1 overexpression increased the length and number of cell protrusions and the relative fluorescence intensity of intracellular filamentous actin (F‐actin), whereas polycystin‐1 knockdown reduced these parameters in response to 15 µg cm^−^^2^ collagen, but not 5 µg cm^−^^2^ collagen. MCF‐7 cells were cultured on slides coated with 5 µg cm^−^^2^ (a) or 15 µg cm^−^^2^ collagen (b). F‐actin and nuclei were stained with rhodamine‐phalloidin (red) and DAPI (blue), respectively. Protrusion length (c), number (d), and intracellular microfilament fluorescence intensity (e) were quantified using confocal microscopy. Arrows indicate cell protrusions. Magnification, 630×. f) Ultracentrifugation‐based fractionation of MCF‐7 cells showed that polycystin‐1 overexpression selectively increased the F‐actin to G‐actin (F/G ratios, F:G) ratio under 15 µg cm^−^^2^ collagen, but not at 5 µg cm^−^^2^ collagen. g–j) Polycystin‐1 overexpression enhanced, whereas its knockdown inhibited, MCF‐7 cell migration (g,h) and invasion (i,j) in response to 15 µg cm^−^^2^ collagen, but not 5 µg cm^−^^2^ collagen. Cells were seeded in Boyden chambers precoated with 5 or 15 µg cm^−^^2^ collagen, with or without Matrigel, and allowed to migrate or invade for 24 h. *n* = 9. Magnification, 100×. *p < 0.05, **p < 0.01, ***p < 0.001, ****p < 0.0001; ns, not significant.

Under 15 µg cm^−^^2^ collagen stimulation, polycystin‐1 overexpression markedly increased the number and length of actin‐based protrusions and the fluorescence intensity of intracellular microfilaments, whereas polycystin‐1 knockdown produced the opposite effects. These changes were not observed under 5 µg cm^−^^2^ collagen (**Figure** **1**a–e; Figure S1e–i, Supporting Information). Furthermore, our results demonstrated that polycystin‐1 overexpression enhanced filamentous actin (F‐actin) assembly (reflected in the ratio of F‐actin to G‐actin; F‐actin, Filamentous actin; G‐actin, globular actin) in MCF‐7 cells under 15 µg cm^−^^2^ collagen, whereas polycystin‐1 knockdown exhibited the opposite effect (Figure 1f). Accordingly, polycystin‐1 overexpression significantly enhanced migration and invasion of both MCF‐7 and MDA‐MB‐231 cells under 15 µg cm^−^^2^ collagen, but had minimal effect under the lower collagen concentration (Figure 1g–j; Figure S2a,b, Supporting Information).

To confirm that polycystin‐1 senses collagen through its LRR domain, we removed the LRR domain from the full‐length polycystin‐1 and assessed cell motility. Boyden chamber assays revealed that the LRR‐deleted polycystin‐1 failed to enhance cell migration and invasion under 15 µg cm^−^^2^ collagen (Figure S2c–f, Supporting Information). Collectively, these results demonstrate that polycystin‐1 mediates collagen‐induced microfilament remodeling and tumor cell motility through its extracellular LRR domain.

### 2.2 Polycystin‐1 Senses ECM Stiffness to Modulate Cytoskeletal Remodeling and Tumor Cell Behavior

**Figure 2:**
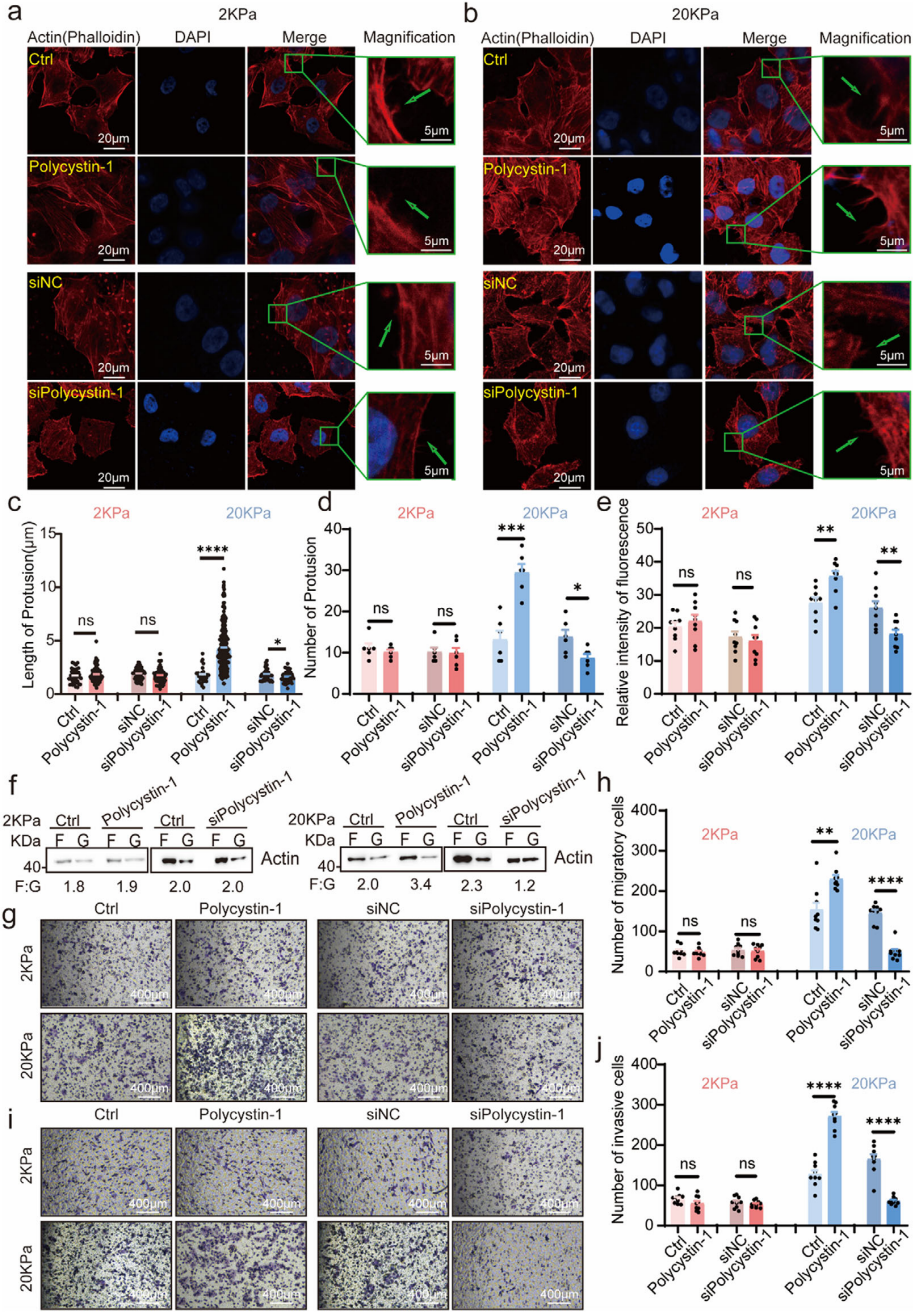
Polycystin‐1 senses ECM stiffness to regulate microfilament remodeling and tumor cell motility. a–e) Polycystin‐1 overexpression increased, while its knockdown reduced the length and number of protrusions and intracellular F‐actin fluorescence intensity in response to 20 kPa PAAG but not 2 kPa PAAG. MCF‐7 cells were cultured on PAAG‐coated slides (2 kPa: a, 20 kPa: b). F‐actin and nuclei were visualized using Rhodamine‐phalloidin and DAPI, respectively. The protrusion length (c), number (d), and fluorescence intensity (e) were measured via confocal microscopy. The arrows indicate cell protrusions. Magnification, 630×. f) Ultracentrifugation‐based fractionation of MCF‐7 cells showed that polycystin‐1 overexpression selectively increased the F‐actin to G‐actin (F/G ratio, F: G) ratio under 20 kPa PAAG collagen conditions, but not at 2 kPa PAAG. g–j) Polycystin‐1 overexpression promoted, while knockdown suppressed, MCF‐7 cell migration (g,h) and invasion (i,j) in response to 20 kPa PAAG, but not 2 kPa PAAG. After 24 h of treatment with 2 or 20 kPa PAAG, the cells were seeded in Boyden chambers and assessed for migration/invasion. *n* = 9. Magnification, 100×. *p < 0.1, **p < 0.01, ***p < 0.001, ****p < 0.0001; ns, not significant.

Excessive collagen deposition increases ECM stiffness, promoting tumor cell motility.^[^ ^12^ ^]^ In an orthologous mouse model of PKD, polycystin‐1 has been shown to respond to stiffness cues from polyacrylamide hydrogels (PAAG) by modulating actomyosin contractility.^[^ ^14^, ^15^ ^]^ To explore whether polycystin‐1 functions as a mechanosensor of ECM stiffness in cancer cells, MCF‐7 and MDA‐MB‐231 cells were exposed to soft (2 kPa) and stiff (20 kPa) PAAG. In cells cultured on 20 kPa PAAG, polycystin‐1 overexpression significantly increased the number and length of actin‐rich protrusions and microfilament fluorescence intensity, while polycystin‐1 knockdown reversed these effects. However, polycystin‐1 manipulation had minimal effects on cells cultured on 2 kPa PAAG (**Figure** **2**a–e; Figure S3a–e, Supporting Information). Similarly, polycystin‐1 overexpression increased the F‐actin to G‐actin ratio (Figure 2f) as well as migration and invasion at 20 kPa PAAG (Figure 2g–j; Figure S3f–i, Supporting Information). These findings indicate that polycystin‐1 senses ECM stiffness and regulates cytoskeletal remodeling and tumor cell motility in a stiffness‐dependent manner. Mechanosignals from both collagen and ECM stiffness produce similar downstream outcomes via polycystin‐1 signaling.

### 2.3 The C‐Terminal Tail of Polycystin‐1 Interacts with the FH2 Domain of Daam1

To elucidate how polycystin‐1 regulates actin cytoskeletal remodeling in response to mechanical cues, we focused on its potential interactions with actin‐nucleating factors. Among these, formins play a central role in the generation of linear actin microfilaments and are crucial mediators of mechanotransduction. In particular, the diaphanous‐related formin Daam1 has been implicated in cytoskeletal remodeling, cell polarity, and directional migration, especially in response to collagen cues.^[^ ^13^ ^]^ Given its established role in actin dynamics and mechanosensitive signaling, we hypothesized that polycystin‐1 may engage Daam1 to relay ECM stiffness signals to the actin cytoskeleton.

**Figure 3:**
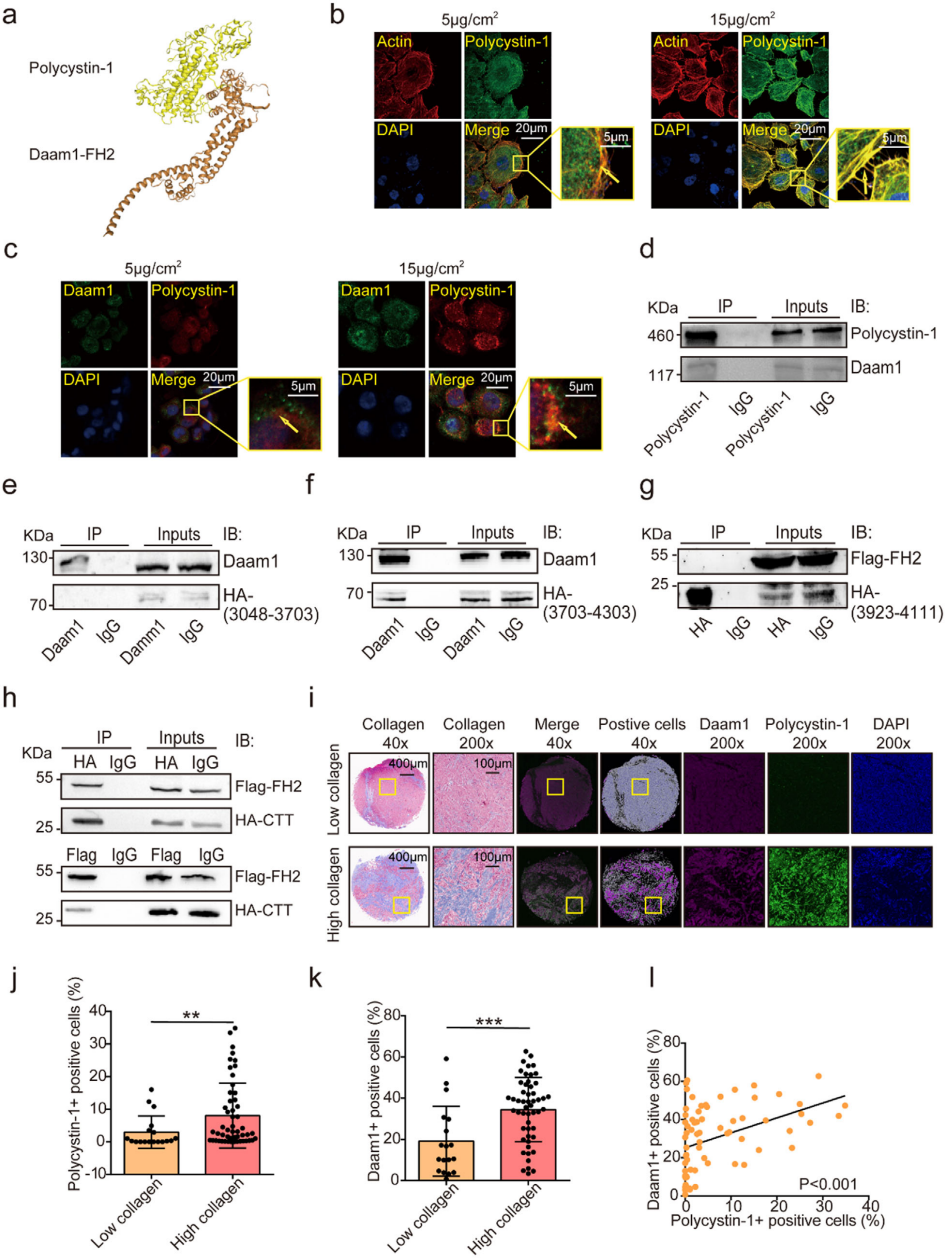
Polycystin‐1 interacts with Daam1. a) The FH2 domain of Daam1 showed the highest binding affinity to polycystin‐1 using the HDOCK server, predicting its binding. Docking score: −233.04. b) Immunofluorescence revealed the co‐localization of polycystin‐1 and F‐actin in MCF‐7 cells treated with 5 or 15 µg cm^−^^2^ collagen. F‐actin was stained with rhodamine and phalloidin. Magnification, 630×. c) Co‐localization of polycystin‐1 and Daam1 in MCF‐7 cells under the same conditions. Magnification, 630×. d) Coimmunoprecipitation showing the interaction between polycystin‐1 and Daam1. HEK‐293T cells overexpressing polycystin‐1 were lysed, and the lysates were immunoprecipitated with anti‐polycystin‐1 and probed with anti‐Daam1. e,f) Daam1 interacts with the intracellular C‐terminal segment (HA‐3703‐4303), but not with the anterior segment (HA‐3048‐3703) of polycystin‐1. g,h) The C‐terminal tail (CTT, 3923–4111) of polycystin‐1 interacts with the FH2 domain of Daam1. Coimmunoprecipitation assays were conducted using HEK‐293T cells expressing HA‐ or FLAG‐tagged constructs. i–l) IHC staining revealed the expression levels (i–k) and correlation (l, *r* = 0.417, Pearson) between polycystin‐1 and Daam1 in breast cancer tissues with varying collagen content. **p < 0.01, ***p < 0.001.

To explore this possibility, we predicted the protein–protein interactions of polycystin‐1 using the HDOCK server and identified Daam1 as a potential binding partner (docking score: −233.04; **Figure** **3**a). Immunofluorescence analysis in MCF‐7 cells revealed co‐localization of polycystin‐1, Daam1, and microfilaments, particularly under 15 µg cm^−^^2^ collagen stimulation (Figure 3b,c). This spatial overlap suggests a functional interaction, which was subsequently confirmed by co‐immunoprecipitation (Co‐IP), demonstrating that polycystin‐1 physically associates with Daam1 (Figure 3d).

To delineate the interaction region, we expressed HA‐tagged intracellular fragments of polycystin‐1: HA‐(3048‐3703) and HA‐(3703‐4303) in HEK‐293T cells (Figure S1a, Supporting Information). The co‐IP results showed that Daam1 interacts with the HA‐(3703‐4303) fragment (Figure 3e,f). Further mapping identified the C‐terminal tail (CTT; residues 4030‐4303) of polycystin‐1 as the region that interacted with the FH2 domain of Daam1 (Figure 3g,h; Figure S1a, Supporting Information). Immunohistochemical analysis of breast cancer tissue microarrays revealed frequent colocalization of polycystin‐1 and Daam1 in highly collagen‐rich areas, with a positive correlation (*r* = 0.417) between their expression levels (Figure 3i–l). These results suggest that polycystin‐1 interacts with Daam1 via its CTT domain, and that both proteins contribute to intracellular microfilament remodeling.

### 2.4 Polycystin‐1 Promotes Microfilament Remodeling and Cell Motility via Daam1

**Figure 4:**
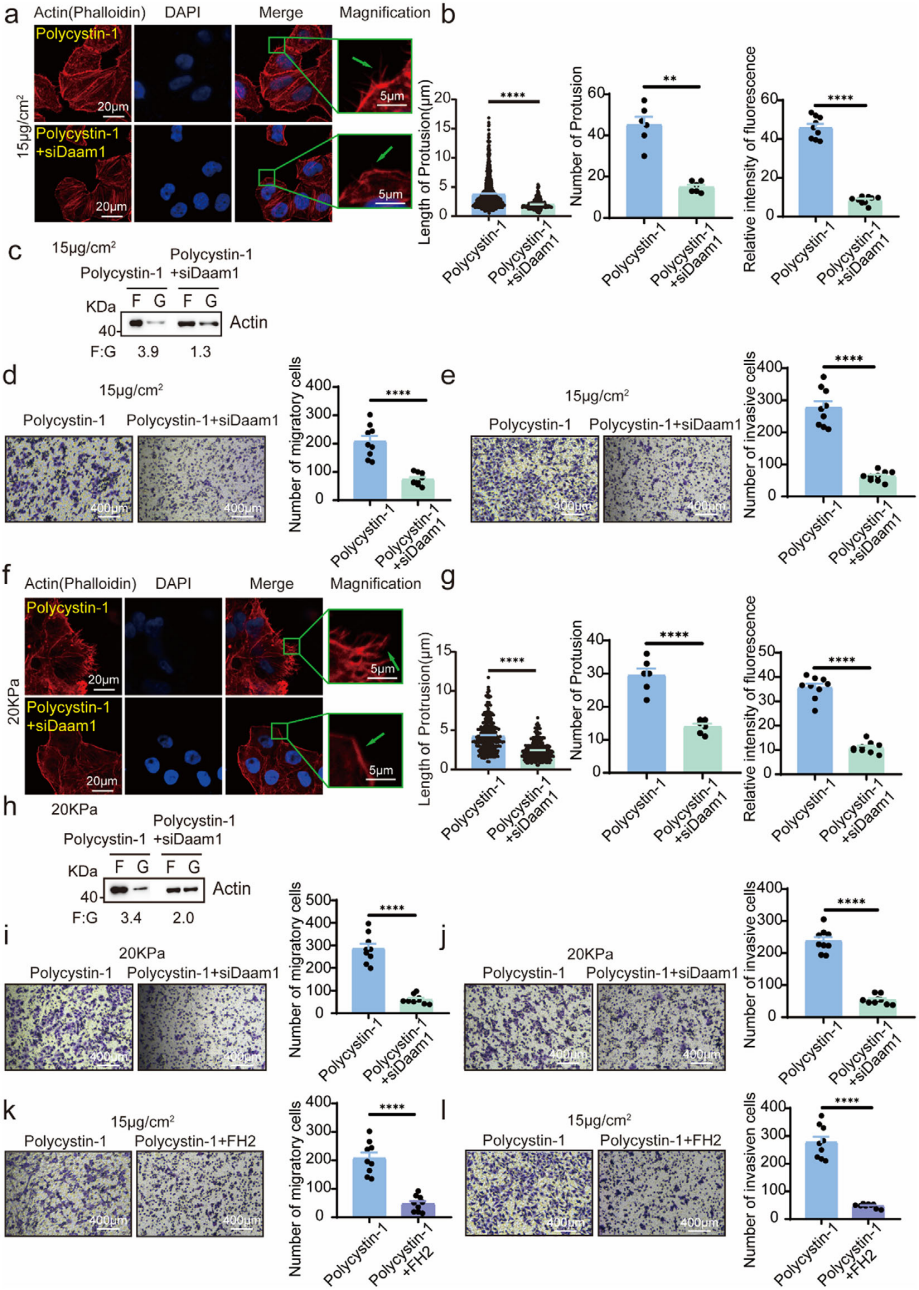
Polycystin‐1 interacts with Daam1 to regulate microfilament remodeling and cell motility. a,b) Co‐overexpression of polycystin‐1 and knockdown of Daam1 reduced collagen‐induced F‐actin polymerization and protrusion formation compared with polycystin‐1 overexpression alone. MCF‐7 cells on 15 µg cm^−^^2^ collagen‐coated slides (a) were stained for F‐actin and nuclei (b–d). Magnification, 630×. c) Co‐overexpression of polycystin‐1 and knockdown of Daam1 reduced the F/G‐actin ratio in MCF‐7 cells compared to polycystin‐1 overexpression alone in response to 15 µg cm^−^^2^ collagen. d,e) Daam1 knockdown attenuated polycystin‐1‐induced cell migration and invasion in response to 15 µg cm^−^^2^ collagen. *n* = 9. Magnification, 100×. f,g) Similar effects were observed on 20 kPa PAAG substrates, with quantification shown in (g). Magnification, 630×. h) Daam1 knockdown attenuated the polycystin‐1‐induced F/G‐actin ratio elevation in 20 kPa PAAG. i,j) Co‐overexpression and Daam1 knockdown inhibited 20 kPa PAAG‐induced migration and invasion. *n* = 9. Magnification, 100×. k,l) Co‐expression of polycystin‐1 and the FH2 domain of Daam1 suppressed 15 µg cm^−^^2^ collagen‐induced migration and invasion. *n* = 9. Magnification, 100×. **p < 0.01, ****p < 0.0001.

To functionally assess the role of the polycystin‐1–Daam1 interaction in microfilament dynamics and cell motility, we overexpressed polycystin‐1 in cells with or without Daam1 knockdown (Figure S4a, Supporting Information). Polycystin‐1 overexpression enhanced the number and length of cell protrusions in response to 15 µg cm^−^^2^ collagen or 20 kPa PAAG, but this effect was abrogated by siRNA‐mediated Daam1 silencing (**Figure** **4**a,b,f,g; Figure S4b,c,f,g, Supporting Information). Daam1 knockdown also attenuated the polycystin‐1‐induced increase in the proportion of F‐actin (Figure 4c,h). Similarly, the polycystin‐1‐induced increases in cell migration and invasion were significantly attenuated by Daam1 knockdown (Figure 4d,e,i,j; Figure S4d,e,h,i, Supporting Information).

Furthermore, overexpression of the FH2 domain of Daam1, which acts as a dominant‐negative decoy, also blocked polycystin‐1‐induced cell migration and invasion (Figure 4k,l; Figure S4j,k, Supporting Information). These data demonstrate that polycystin‐1 modulates microfilament remodeling and tumor cell motility in response to collagen and ECM stiffness through its interaction with Daam1.

### 2.5 Polycystin‐1/Daam1/RhoA Axis Drives Microfilament Remodeling and Tumor Cell Motility

**Figure 5:**
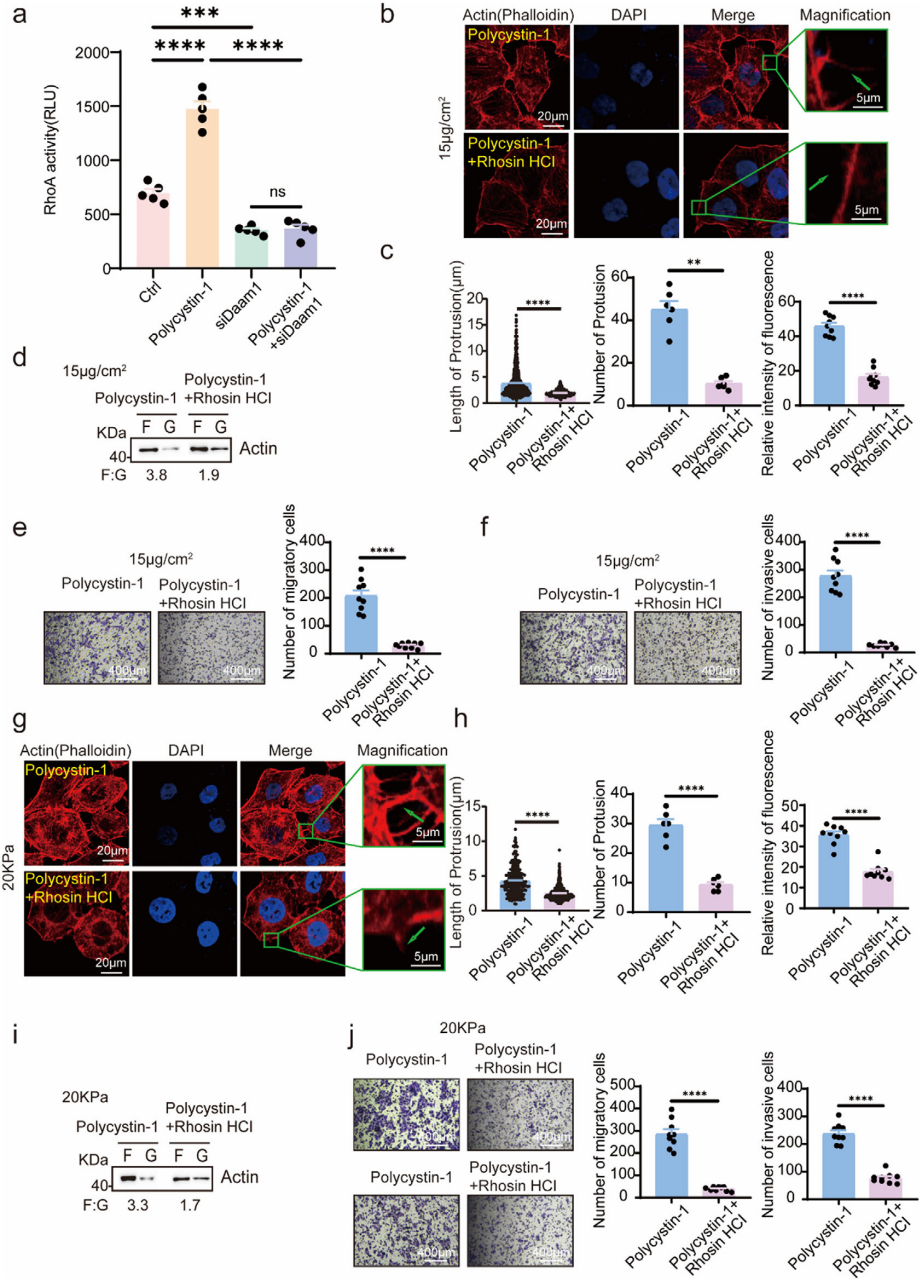
Polycystin‐1/Daam1 activates RhoA to regulate microfilament dynamics and motility. a) RhoA activity in collagen‐treated HEK‐293T cells was quantified by G‐LISA and analyzed by one‐way analysis of variance. b,c) Rhosin HCl, a RhoA inhibitor, suppressed polycystin‐1‐induced actin polymerization in MCF‐7 cells on 15 µg cm^−^^2^ collagen. F‐actin and nuclei are stained (b), and the protrusion metrics are shown. Magnification, 630×. d) Rhosin HCl inhibited polycystin‐1‐induced F/G‐actin ratio elevation in MCF‐7 cells in response to 15 µg cm^−^^2^ collagen. e,f) Rhosin HCl reduced polycystin‐1‐induced migration and invasion. n = 9. Magnification, 100×. g,h) Similar inhibitory effects were observed with the 20 kPa PAAG treatment (g), as shown by quantification (h). i) Rhosin HCl inhibited polycystin‐1‐induced F/G‐actin ratio elevation at 20 kPa PAAG. j) Rhosin HCl blocked 20 kPa PAAG‐induced migration (top) and invasion (bottom) in polycystin‐1 overexpressing cells. *n* = 9. Magnification, 100×. **p < 0.01, ***p < 0.001, ****p < 0.0001; ns, not significant.

Given our previous findings that Daam1 activates RhoA to regulate actin polymerization and cell migration,^[^ ^16^, ^17^, ^18^ ^]^ we assessed whether polycystin‐1 signals through the Daam1/RhoA pathway. G‐LISA assay results revealed that polycystin‐1 overexpression increased RhoA activity in collagen‐treated HEK‐293T cells, whereas Daam1 knockdown suppressed this effect (**Figure** **5**a).

Pharmacological inhibition of RhoA using rhosin hydrochloride significantly reduced the number and length of actin‐rich protrusions and decreased microfilament fluorescence intensity in cells treated with 15 µg cm^−^^2^ collagen or 20 kPa PAAG (Figure 5b,c,g,h; Figure S5a,b,e,f, Supporting Information) and a polycystin‐1‐induced increase in the proportion of F‐actin (Figure 5d,i). Rhosin hydrochloride also attenuated polycystin‐1‐induced cell migration and invasion (Figure 5e,f,j; Figure S5c,d,g,h, Supporting Information). These results confirm that polycystin‐1 transduces mechanosignals via the Daam1/RhoA axis to regulate cytoskeletal remodeling and tumor cell motility.

### 2.6 Single‐Molecule Force Spectroscopy Analysis of the GPS Domain of Polycystin‐1 and its R3039H Mutant

Polycystin‐1 undergoes autocatalytic cleavage at the G protein‐coupled receptor proteolytic site (GPS) domain, and the L3048H mutation within this domain has been reported to reduce susceptibility to cleavage in PKD.^[^ ^19^, ^20^ ^]^ We hypothesized that the impaired cleavage of polycystin‐1 may influence its mechanotransductive properties in tumor cells. To test this hypothesis, we queried the UniProt database for GPS domain mutations associated with breast cancer and identified R3039H as a recurrent mutation (Table S1, Supporting Information). We further screened the GPS domain‐coding region in 131 breast cancer specimens and found that the c.9116G>A (p.R3039H) mutation occurred in 22 cases. Clinicopathological correlation analysis revealed that the R3039H variant was significantly associated with an increased risk of lymphatic and distant metastases (**Table** **1**).

**Table 1:** Associations between polycystin‐1 c.9116G>A (p.R3039H) and clinicopathological characteristics in breast cancer.

Variables	Genotype	OR (95% CI)	*p* value
tumor size	GG (9/93)	—	—
(>4 cm/≤4 cm)	GA (1/19)	0.2574–21.15	>0.9999
lymphatic metastasis	GG (17/92)	—	—
(yes/no)	GA (8/14)	0.1149–0.9238	**0.0496**
distant metastasis	GG (2/107)	—	—
(yes/no)	GA (4/18)	0.01562–0.3946	**0.0074**
ER state	GG (67/4)	—	—
(positive/negative)	GA (14/1)	0.09181–8.347	>0.9999
PR state	GG (59/9)	—	—
(positive/negative)	GA (12/3)	0.1495–2.353	0.4473
Her‐2 state	GG (40/16)	—	—
(positive/negative)	GA (7/5)	0.1532–1.896	0.3729
P53 state	GG (23/40)	—	—
(positive/negative)	GA (2/7)	0.09861–2.681	0.4819
Ki67 state	GG (35/66)	—	—
(positive/negative)	GA (9/11)	0.6133–4.186	0.3795

Differences were analyzed using chi‐square or Fisher's exact test.

**Figure 6:**
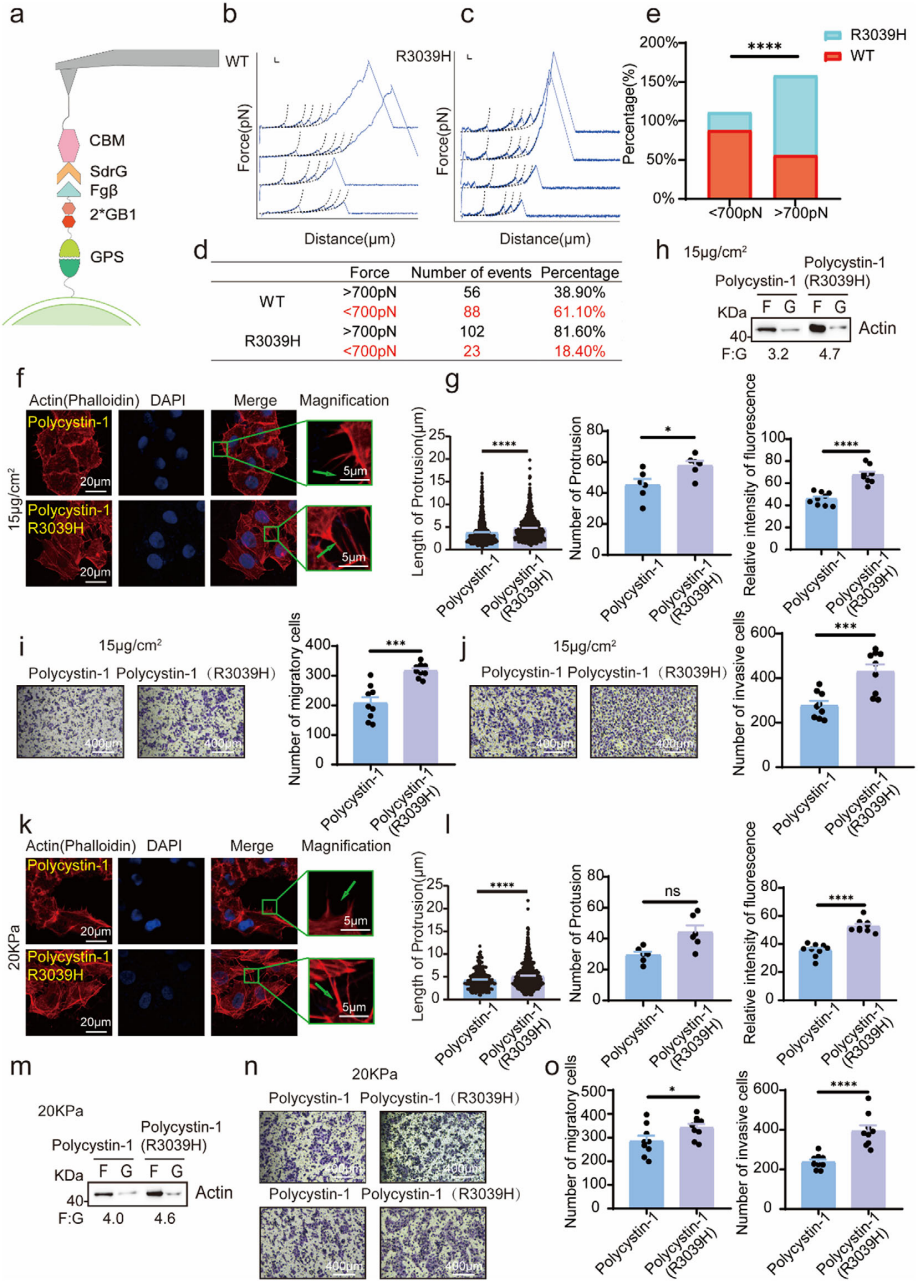
Force spectroscopy of the GPS domain of polycystin‐1 and its impact on microfilament remodeling and cell motility. a) Schematic of atomic force microscopy‐based single‐molecule force spectroscopy (AFM‐SMFS), with domains including GPS, GB1, Fgβ/SdrG, CBM, and AFM tip. b,c) SMFS analysis of GPS domains in the polycystin‐1 WT (b) and R3039H mutant (c). d,e) Cleavage events of GPS domains analyzed by chi‐square test. f,g) Polycystin‐1 R3039H enhanced collagen‐induced actin polymerization compared to the WT. MCF‐7 cells on 15 µg cm^−^^2^ collagen were stained (f) and analyzed (g). Magnification, 630×. h) R3039H increased the F/G‐actin ratio in MCF‐7 cells in response to 15 µg cm^−^^2^ collagen compared to WT. i,j) R3039H promoted cell migration (i) and invasion (j) in response to 15 µg cm^−^^2^ collagen. *n* = 9. Magnification, 100×. k,l) Similar enhancements were observed on the 20 kPa PAAG substrates (k), as quantified in (l). Magnification, 630×. m) R3039H increased the F/G‐actin ratio in MCF‐7 cells in response to 20 kPa PAAG. n,o) R3039H increased migration (top) and invasion (bottom) upon stimulation with 20 kPa PAAG. *n* = 9. Magnification, 100×. *p < 0.05, ***p < 0.001, ****p < 0.0001; ns, not significant.

Atomic force microscopy‐based single‐molecule force spectroscopy (AFM‐SMFS) was employed to assess the cleavage susceptibility of polycystin‐1 at the single‐molecule level. For reliable detection, polycystin‐1 constructs were engineered with two GB1 domains and an Fgβ tag and immobilized on a surface as described previously^[^ ^21^ ^]^ (**Figure** **6**a). Then, an AFM tip coated with CBM domain and SdrG approached the surface and picked up polycystin‐1 by forming a Fgβ‐SdrG interaction. Thus, Fgβ‐SdrG served as the handle, and the less stable protein domains (CBM and GB1), with unfolding forces lower than ≈200 pN, served as single‐molecule fingerprints. Upon stretching, all proteins were unfolded, leading to a sawtooth‐like force‐extension curve (Figure 6b,c; Figure S6a,b, Supporting Information). An AFM cantilever tip functionalized with a CBM domain and SdrG protein was used to capture the protein via specific Fgβ–SdrG interactions. The CBM and GB1 domains served as force calibration markers because their unfolding forces are typically <200 pN. Upon extension, a characteristic sawtooth force‐extension pattern was generated as the domains unfolded sequentially (Figure 6b,c; Figure S6a,b, Supporting Information). The final force peak corresponded to the rupture of the most stable domain. ≈60% of wild‐type (WT) polycystin‐1 molecules exhibited rupture forces below 700 pN, whereas ≈20% of the R3039H or L3048H mutants showed such low‐force rupture events (Figure 6d,e; Figure S6c,d, Supporting Information). Given that effective mechanotransduction requires a stable protein backbone for force propagation, these findings suggest that the R3039H mutation renders polycystin‐1 more resistant to cleavage, thereby enhancing their capacity for force transmission.

Next, we evaluated the functional consequences of the R3039H mutation on cytoskeletal remodeling and cell motility. Polycystin‐1 WT and R3039H constructs were overexpressed in MCF‐7 cells (Figure S6e, Supporting Information). Cells expressing the R3039H variant displayed enhanced sensitivity to collagen and PAAG stiffness, evidenced by longer and more abundant protrusions and increased F‐actin fluorescence intensity (Figure 6f,g,k,l; Figure S6f,g,j,k, Supporting Information), and the proportion of F‐actin (Figure 6h,m). Moreover, R3039H overexpression significantly increased tumor cell migration and invasion (Figure 6i,j,n,o; Figure S6h,i,l,m, Supporting Information) compared with WT polycystin‐1.

### 2.7 Polycystin‐1 R3039H Promotes YAP Nuclear Translocation and Upregulates CTGF Expression

**Figure 7:**
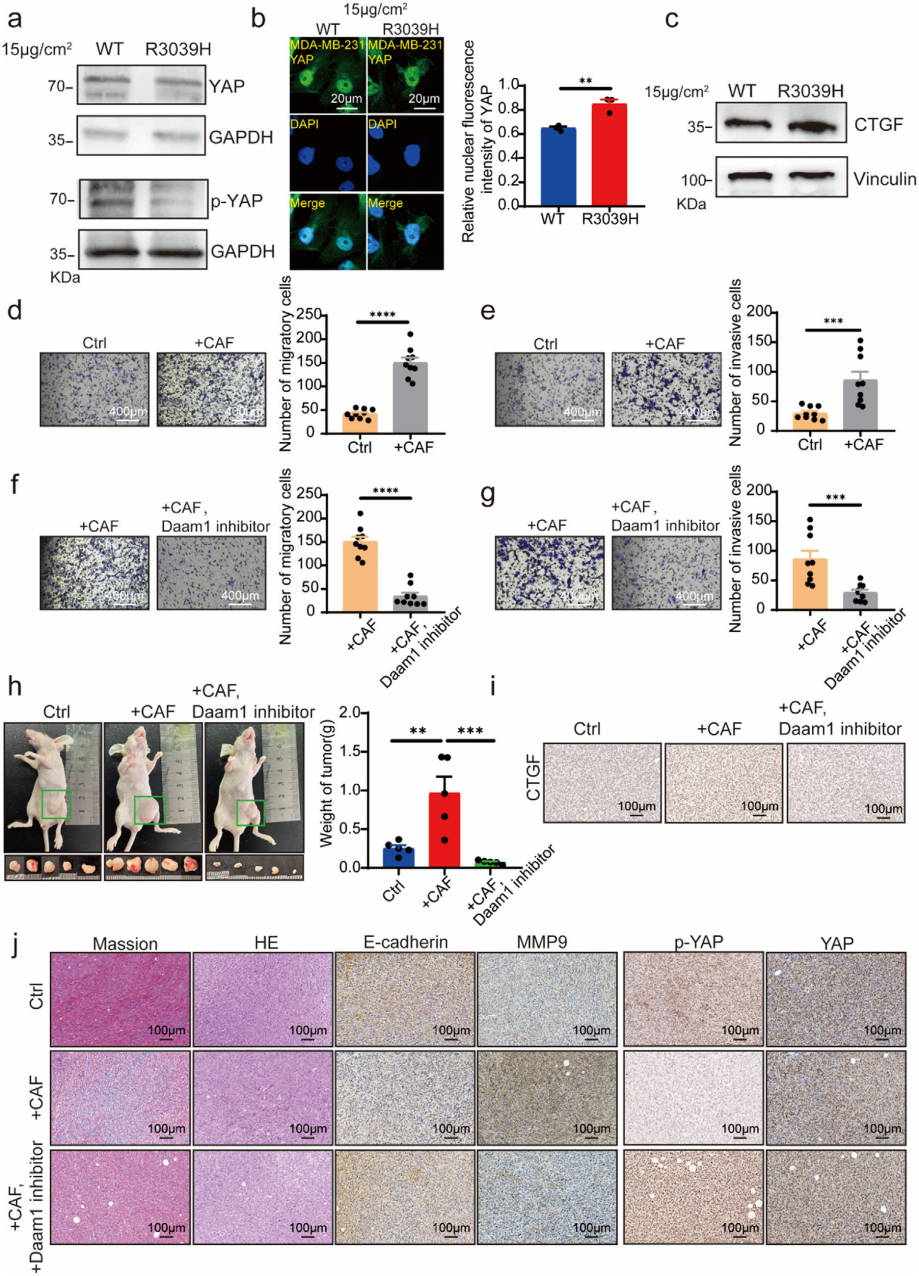
Polycystin‐1 R3039H promotes CTGF expression by facilitating nuclear translocation of YAP. a) Overexpression of polycystin‐1 R3039H decreased the phosphorylation of YAP at Ser127 (p‐YAP S127) in response to 15 µg cm^−^^2^ collagen, compared with polycystin‐1 WT. MDA‐MB‐231 cells were transiently transfected with polycystin‐1 WT or R3039H constructs. Whole‐cell lysates were subjected to immunoblotting using anti‐YAP and anti‐p‐YAP (S127) antibodies. Representative data from one of three independent experiments are shown. b) Overexpression of polycystin‐1 R3039H enhanced nuclear translocation of YAP in response to 15 µg cm^−^^2^ collagen, compared with WT. YAP localization was detected by immunofluorescence using an anti‐YAP1 antibody. Magnification, 630×. c) Polycystin‐1 R3039H increased CTGF expression in response to 15 µg cm^−^^2^ collagen compared to WT. Whole‐cell lysates were analyzed by immunoblotting with anti‐CTGF antibody. Representative data from one of three independent experiments are shown. d,e) Co‐culture with cancer‐associated fibroblasts (CAFs) promotes migration (d) and invasion (e) of MDA‐MB‐231 cells. MDA‐MB‐231 cells and CAFs were seeded into the upper and lower chambers of the Boyden inserts, respectively. The cells were allowed to migrate or invade for 24 h. *n* = 9. Magnification, 100×. f,g) Treatment with the Daam1 inhibitor etoposide (270 µm) suppressed the CAF‐induced migration (f) and invasion (g) of MDA‐MB‐231 cells in co‐culture assays. *n* = 9. Magnification, 100×. h) CAFs promoted subcutaneous tumor growth in nude mice, whereas injection of the Daam1 inhibitor etoposide suppressed tumor progression. Mice were injected subcutaneously with 5 × 10⁶ MDA‐MB‐231 cells alone (Ctrl), or co‐injected with 5 × 10⁶ MDA‐MB‐231 cells and 5 × 10⁶ CAFs (+CAF and +CAF, Daam1 inhibitor groups). From day 7, mice in the +CAF and +CAF, Daam1 inhibitor groups received daily subcutaneous injections of etoposide for seven consecutive days. Tumor volume was analyzed using one‐way analysis of variance. i) CAFs enhanced CTGF expression in xenograft tumors, which was reduced by etoposide treatment. Tumor tissues were harvested one month post‐injection and stained with an anti‐CTGF antibody. Magnification, 200×. j) Compared to the control groups, CAFs showed reduced E‐cadherin expression, increased MMP9, and reduced p‐YAP S127 levels, with no change in total YAP levels. However, these effects were reversed by etoposide treatment. Serial tumor sections were stained with antibodies against E‐cadherin, MMP9, p‐YAP, and YAP. Masson's trichrome staining was used to assess collagen deposition. Magnification, 200×. **p < 0.01, ***p < 0.001, **p < 0.0001.

Yes‐associated protein (YAP) is a transcriptional effector that translocates to the nucleus in response to ECM stiffness.^[^ ^22^, ^23^ ^]^ Its activity is regulated via RhoA signaling, where phosphorylated YAP (p‐YAP) is retained in the cytoplasm, preventing its entry into the nucleus.^[^ ^24^, ^25^, ^26^ ^]^ Polycystin‐1 mutations have been shown to activate the RhoA/YAP/c‐Myc axis, contributing to autosomal dominant polycystic kidney disease (ADPKD) progression.^[^ ^27^ ^]^ In mouse models of ADPKD, the nuclear accumulation of YAP has been observed in cystic epithelial cells and dilated renal tubules.^[^ ^28^ ^]^ In our study, overexpression of polycystin‐1 R3039H significantly reduced p‐YAP (Ser127) levels (**Figure** **7**a; Figure S7a, Supporting Information) and promoted the nuclear translocation of YAP (Figure 7b; Figure S7b, Supporting Information) in tumor cells cultured on 15 µg cm^−^^2^ collagen.

As a transcriptional coactivator, YAP enhances the expression of connective tissue growth factor (CTGF),^[^ ^29^, ^30^ ^]^ which subsequently activates cancer‐associated fibroblasts (CAFs) and induces collagen secretion.^[^ ^31^, ^32^ ^]^ We observed significant upregulation of CTGF expression upon polycystin‐1 R3039H overexpression (Figure 7c; Figure S7c, Supporting Information), and exogenous CTGF treatment further elevated collagen I levels (Figure S7d, Supporting Information). Co‐culture of tumor cells with CAFs enhanced tumor cell migration (Figure 7d; Figure S7e, Supporting Information) and invasion (Figure 7e; Figure S7f, Supporting Information). Notably, the Daam1 inhibitor etoposide^[^ ^33^ ^]^ substantially blocked the polycystin‐1 R3039H‐induced increase in cell migration (Figure 7f; Figure S7g, Supporting Information) and invasion (Figure 7g; Figure S7h, Supporting Information).

In a mouse xenograft model, MDA‐MB‐231 cells were co‐injected with CAFs in a 1:1 ratio; control mice received tumor cells only. Tumor weights in the +CAF group were significantly higher than those in the control group, whereas etoposide treatment significantly reduced the tumor burden (Figure 7h). CTGF levels were markedly lower in the +CAF and Daam1 inhibitor groups than in the +CAF group alone (Figure 7i). Immunohistochemistry revealed that the +CAF group exhibited an elevated expression of matrix metalloproteinase‐9 (MMP‐9), reduced E‐cadherin levels, and decreased p‐YAP levels, whereas total YAP expression remained unchanged (Figure 7j), indicating enhanced invasiveness. However, etoposide treatment reversed these changes (Figure 7j).

Collectively, these findings suggest that membrane‐localized polycystin‐1 senses ECM stiffness and promotes CTGF expression via Daam1‐dependent signaling. Elevated CTGF then activates CAFs, which secrete collagen, remodel the ECM, and promote tumor cell motility and invasion.

## 3 Discussion

The polycystin family consists of two members: polycystin‐1 and polycystin‐2. Polycystin‐2 functions as a calcium‐permeable ion channel.^[^ ^34^ ^]^ Polycystin‐1 senses fluid shear stress, such as that generated by blood or urinary flow, and subsequently activates polycystin‐2 to mediate calcium influx into cardiomyocytes and renal epithelial cells.^[^ ^35^, ^36^ ^]^ In this study, we demonstrate that polycystin‐1 serves as a mechanosensor that transduces ECM stiffness and collagen‐derived mechanical signals to the intracellular cytoskeleton, ultimately regulating tumor cell motility.

Polycystin‐1 is a large (≈460 kDa) plasma membrane protein characterized by an extensive extracellular N‐terminal domain (containing an LRR domain), 11 transmembrane segments, and a short intracellular C‐terminal tail (CTT).^[^ ^37^, ^38^, ^39^ ^]^ Collagen binds directly to the LRR domain of polycystin‐1.^[^ ^9^ ^]^ Loss‐of‐function mutations in the *PKD1* gene, which encodes polycystin‐1, are the major cause of ADPKD.^[^ ^38^, ^39^ ^]^ Emerging evidence also implicates polycystin‐1 in cancer metastasis.^[^ ^40^, ^41^, ^42^, ^43^, ^44^ ^]^ Interestingly, polycystin‐1 exhibits cell‐context‐dependent effects; it suppresses migration in GOS3 glioma cells but enhances migration in A549 non‐small cell lung cancer cells. Similarly, polycystin‐1 inhibits the proliferation of GOS3 cells and promotes the proliferation of MCF‐7, A549, and HT‐29 cells.^[^ ^42^ ^]^ Here, we revealed a novel function of polycystin‐1 in modulating tumor cell motility by linking extracellular mechanical cues with intracellular microfilament dynamics.

Under physiological conditions, polycystin‐1 undergoes autoproteolytic cleavage of the GPS domain, specifically at HL^T, residue 3048. The resulting N‐ and C‐terminal fragments remain associated via noncovalent interactions. The L3048H mutation, which is frequently observed in ADPKD, reduces the susceptibility of polycystin‐1 to GPS cleavage.^[^ ^19^, ^45^, ^46^, ^47^, ^48^, ^49^ ^]^ We hypothesized that this mutation confers enhanced mechanosensitivity in response to extracellular collagen. Using AFM‐SMFS, we provide direct biophysical evidence that polycystin‐1 L3048H exhibits impaired cleavage in vitro.

Solid tumors are subjected to various mechanical forces, including compression and tension, resulting from uncontrolled cell proliferation.^[^ ^50^ ^]^ These forces contribute to aberrant mechanotransduction, cytoskeletal disruption, and increased malignancy.^[^ ^12^, ^51^, ^52^ ^]^ A key source of these forces is the collagen‐rich ECM,^[^ ^10^, ^53^ ^]^ which undergoes stiffening due to fibrosis, a hallmark of many solid tumors.^[^ ^54^, ^55^, ^56^, ^57^ ^]^ Elevated ECM stiffness promotes tumor progression and metastasis.^[^ ^54^, ^58^, ^59^, ^60^, ^61^, ^62^ ^]^ Mechanical mapping of the tumors revealed a stiff periphery (≈20 kPa) and a softer core (≈2 kPa). We recapitulated this mechanical heterogeneity using PAAGs of varying stiffness (2 kPa vs 20 kPa), demonstrating that a stiff ECM facilitates microfilament remodeling and cell motility in a polycystin‐1 mutation‐dependent manner.

Previous studies have shown that polycystin‐1 regulates the cytoskeletal organization and migration of renal epithelial cells.^[^ ^63^, ^64^ ^]^ However, the mechanisms underlying polycystin‐1‐mediated cytoskeletal regulation in tumor cells remain unclear. Actin‐rich membrane protrusions are critical for directional migration^[^ ^65^ ^]^ and metastatic dissemination,^[^ ^66^, ^67^ ^]^ and stiff microenvironments enhance the formation of these protrusions in breast cancer cells.^[^ ^10^ ^]^

In the present study, we identified Daam1 as a key downstream effector that interacts with the CTT of polycystin‐1. Daam1, which organizes actin structures along the pseudopod axis,^[^ ^68^ ^]^ mediates the transmission of mechanical signals from high collagen concentrations and stiff ECM to the actin cytoskeleton, thereby promoting protrusion formation, migration, and invasion. We have previously demonstrated that Daam1 activates RhoA to regulate cytoskeletal remodeling.^[^ ^17^ ^]^ Here, we show that polycystin‐1 upregulation enhances Daam1/RhoA signaling, resulting in increased microfilament remodeling and membrane protrusions.

YAP activity is mechanosensitive and is tightly regulated by Rho GTPase‐mediated actomyosin contractility in response to ECM stiffness.^[^ ^22^, ^24^ ^]^ Consistent with this, we observed that the tumor‐associated polycystin‐1 R3039H mutant showed reduced YAP phosphorylation and promoted its nuclear localization, indicating increased YAP activity under stiff ECM conditions. In the nucleus, YAP transcriptionally upregulates CTGF, a key mediator of matrix remodeling and fibrosis. Elevated CTGF expression can activate CAFs, enhance collagen production, and reinforce ECM stiffness.^[^ ^30^, ^31^ ^]^ This establishes a feed‐forward loop that amplifies mechanical signaling through polycystin‐1 and further activates YAP. Importantly, YAP activation has been mechanistically linked to epithelial–mesenchymal transition (EMT) through the repression of epithelial markers, such as E‐cadherin, and the induction of mesenchymal traits. In our study, polycystin‐1 R3039H‐induced YAP activation coincided with reduced E‐cadherin levels, suggesting that polycystin‐1 promotes the mesenchymal phenotype via the YAP/CTGF axis. Thus, the polycystin‐1/RhoA/YAP pathway not only mediates tumor cell mechanotransduction but also facilitates EMT by coupling mechanical cues to transcriptional programs that drive invasiveness.

In conclusion, our findings establish polycystin‐1 as a key transducer of extracellular mechanical signals, sensing both collagen engagement and ECM stiffness to regulate cytoskeletal dynamics and tumor cell motility. The cleavage‐resistant R3039H mutant enhances this mechanosignaling function. Moreover, we demonstrate that polycystin‐1‐mediated mechanotransduction activates Daam1/RhoA signaling and facilitates microfilament remodeling. Polycystin‐1 R3039H‐induced YAP nuclear translocation leads to increased CTGF expression and collagen deposition by CAFs. These results highlight a novel mechanism that governs tumor cell motility through mechanosensitive signaling and provide new insights into the mechanical regulation of cancer progression.

## 4 Experimental Section

### Clinical Samples

A total of 131 breast cancer tissue samples were collected between 2021 and 2023 at the First Affiliated Hospital of Nanjing Medical University and the Affiliated Wuxi People's Hospital of Nanjing Medical University. All patients were pathologically diagnosed with breast cancer using hematoxylin and eosin staining. Ethical approval for this study was obtained from the Clinical Research Ethics Committee of Nanjing Medical University (No. 2020‐93) and written informed consent was obtained from all participants.

### Western Blot Analysis

Cultured cells (MCF‐7, MDA‐MB‐231, HEK‐293T, and CAFs) were washed with phosphate‐buffered saline (PBS) and lysed in RIPA buffer (Beyotime, P10013B/C) supplemented with protease inhibitors. Lysates were collected and proteins were separated by sodium dodecyl sulfate‐polyacrylamide gel electrophoresis (SDS‐PAGE) (Epizyme, PG112, PG113), followed by transfer onto nitrocellulose membranes (Millipore, HATF00010). The membranes were incubated with primary antibodies against polycystin‐1 (Santa Cruz, sc‐130554), Daam1 (Proteintech, 14876‐1‐AP, 1:1000), GAPDH (Proteintech, 10494‐1‐AP, 1:10000), FLAG (Proteintech, 20543‐1‐AP, 1:10000), HA (Proteintech, 51064‐2‐AP, 1:1000), phospho‐YAP (S127, Abclonal, AP1436, 1:1000), YAP (Proteintech, 13584‐1‐AP, 1:1000), CTGF (Santa Cruz, sc‐565970, 1:500), and COL1a1 (Proteintech, 67288‐1‐Ig, 1:1000). After incubation with the appropriate secondary antibodies, signals were detected using enhanced chemiluminescence.

For polycystin‐1 analysis, cells were lysed using a modified protein lysis buffer to ensure extraction of the full‐length protein. SDS‐PAGE was performed under low voltage (20 V) in an ice‐water bath for 13 h to allow the proper separation of high‐molecular‐weight proteins. Proteins were then transferred onto membranes at a constant current of 300 mA in an ice‐water bath for 10–13 h. The primary antibody against polycystin‐1 was incubated at 4 °C for at least 2 days to improve detection sensitivity.

### Cell Culture and Reagents

MCF‐7, MDA‐MB‐231, and HEK‐293T cell lines, which were verified to be free of microbial contamination, were obtained from the National Collection of Authenticated Cell Cultures (Shanghai, China). These cell lines were selected to represent breast cancer cells with different levels of aggressiveness (MCF‐7: luminal‐like, less invasive; MDA‐MB‐231: basal‐like, highly invasive), and for plasmid transfection and protein overexpression assays (HEK‐293T). Primary CAFs were isolated from breast tumor tissues to recapitulate the tumor microenvironment. MDA‐MB‐231 cells were cultured in L‐15 medium (KeyGEN BioTECH, KGM41300N‐500), while MCF‐7, HEK‐293T, and CAFs were maintained in Dulbecco's Modified Eagle Medium (Gibco, 8123393), all supplemented with 10% fetal bovine serum (FBS; ExCell, FSP500) and 1% penicillin/streptomycin (Gibco, 15140122), at 37 °C in a 5% CO_2_ humidified incubator.

The RRIDs of relevant reagents are provided in Table S2 (Supporting Information).

### Collagen Coating

Collagen coating of 24‐well and six‐well plates, and 60 mm dishes was performed using a solution prepared by diluting collagen (Gibco, A10483‐01) to 0.1 mg mL^−1^ in sterile 0.006 mol L^−1^ acetic acid. Subsequently, a volume corresponding to 60% of the final culture volume was added to each dish. After allowing the coating to air‐dry for 1 h in a sterile environment, final collagen concentrations of 5, 15, and 25 µg cm^−^^2^ were achieved.

### Polyacrylamide Hydrogels

Hydrogels were prepared as previously described. Briefly, 15 mm circular coverslips were treated sequentially with 0.1 M NaOH, 3‐aminopropyltriethoxysilane (APES, Macklin, A800523), and 0.5% glutaraldehyde (Sigma–Aldrich, 111‐30‐8). Glass slides were treated with 100 µL DCDMS (Macklin, D806824). Polyacrylamide and bis‐acrylamide (Beyotime, ST004; Macklin, 110‐26‐9) were mixed in varying ratios (**Table** **2**) to obtain different stiffness levels and polymerized for 10 min using ammonium persulfate and TEMED at 1:100 and 1:1000 ratios, respectively. The hydrogels were soaked in PBS at 4 °C for 24 h. One day before use, the gels were activated with 0.2 mg mL^−1^ sulfo‐SANPAH (Thermo Fisher, 22589) under 365 nm UV for 10 min and subsequently coated with HEPES‐buffered collagen I (HEPES 50 mm, pH 8.5, Sigma–Aldrich, H3375; collagen I, Gibco, A10483‐01). The gels were washed three times with PBS and UV‐irradiated for 30 min before cell seeding.

**Table 2:** Composition of polyacrylamide hydrogels.

Stiffness	40% Arc‐Bis /µL	2% Bis‐Acrylamide /µL	ddH_2_O /µL	Total volume /µL
2 KPa	200	24	776	1000
20 KPa	200	132	668	1000

Plasmid construction and transfection.

The truncated FH2 domain of Daam1 was PCR‐amplified from a full‐length Daam1 plasmid (lab stock) and cloned into pcDNA containing a FLAG tag (600–1009 bp). The full‐length polycystin‐1 plasmid was obtained from Addgene (pCI‐PC1‐Flag, #21369), and mutant forms (R3039H, LRR‐deleted) were synthesized using GenScript. Truncated intracellular domains were generated from pCI‐PC1‐Flag and tagged with HA (HA‐3048‐3703, 3703‐4303, 3923‐4111, CTT). The GPS‐truncated and mutant constructs (L3048H, R3039H) were synthesized using GenScript. siRNAs targeting Daam1 (5′‐TTAGATTGAGAACACTGGG‐3′) and polycystin‐1 (5′‐CAAGACAUUAUUGCCGAGCUTT‐3′) were synthesized by Shanghai GenePharma. Transfection was performed using Lipofectamine 3000 (Invitrogen, L3000075) when the cells reached 80–90% confluence and incubated for 48 h.

### F‐Actin/G‐Actin Fractionation

The cells were rinsed with PBS and lysed in warm LAS2 buffer (according to the manufacturer's instructions; cytoskeleton, BK037). The lysates were homogenized using a motorized homogenizer and incubated at 37 °C for 10 min. After incubation, 100 µL of each lysate was collected and centrifuged at 350 × *g* for 5 min at room temperature to remove unbroken cells or debris. The resulting supernatants were transferred to ultracentrifuge tubes and centrifuged at 100 000 × *g* for 1 h at 37 °C to separate F‐actin (pellet) from G‐actin (supernatant). The supernatants were carefully transferred to fresh tubes. F‐actin pellets were resuspended in 100 µL of F‐actin depolymerization buffer and incubated on ice for 1 h with pipetting every 15 min. Finally, 25 µL of 5× SDS sample buffer was added to both the pellet and supernatant fractions. Samples were resolved by SDS‐PAGE and analyzed by western blotting using the appropriate antibodies.

### Boyden Chamber Assays

MCF‐7 and MDA‐MB‐231 cells were seeded on collagen‐ or PAAG‐coated 24‐well plates, transfected with siRNAs or plasmids, and allowed to reach 80–90% confluence. For invasion assays, the upper chambers of Transwell inserts (8.0 µm, Labselect, 14341) were precoated with Matrigel (Corning, 356234). A single‐cell suspension (1 × 10⁶ cells) in serum‐free medium was added to the upper chamber, and the lower chamber contained 20% FBS medium. After 24 h at 37 °C, non‐invading cells were removed, and the membranes were fixed with 4% paraformaldehyde, stained with 0.5% crystal violet, and imaged microscopically.

### Immunofluorescence and Microfilament Staining

Cells cultured in collagen‐ or PAAG‐coated 24‐well plates were fixed in 4% paraformaldehyde for 20 min, permeabilized, and blocked (Coolaber, SL1336) for 1 h. The cells were incubated overnight at 4 °C with primary antibodies against polycystin‐1 (sc‐130554, 1:50; Santa Cruz Biotechnology), Daam1 (14876‐1‐AP, 1:1000; Proteintech), and YAP (13584‐1‐AP, 1:1000; Proteintech), followed by FITC‐labeled secondary antibodies (Sigma‐Aldrich). Microfilaments were stained with rhodamine‐phalloidin (Yesen, 40734ES75) for 1 h, and nuclei counterstained with DAPI (SouthernBiotech, 010‐20). Images were acquired with a Zeiss LSM710 confocal microscope and analyzed using the ZEN software.

### Co‐Immunoprecipitation (Co‐IP)

Cells were lysed in IP lysis buffer (Pierce, 87787), and lysates were incubated overnight at 4 °C with target antibodies. Protein A/G agarose beads (Pierce, 20421) were added and incubated for 2 h at room temperature (22–24 °C). The beads were washed and eluted with SDS sample buffer (Cwbio, 01411), followed by western blotting using specific antibodies, including anti‐FLAG (Proteintech, 66008‐4‐Ig) and anti‐HA (Abcam, ab236632).

### Tissue Microarray and Multiple Immunohistochemistry (mIHC)

A breast cancer tissue microarray (HBreD090pg01) containing 70 samples and the associated clinicopathological data were purchased from OutDo BioTECH (Shanghai, China). Ethical approval was obtained from the Clinical Research Ethics Committee of OutDo BioTECH. Masson's trichrome, Sirius Red, and mIHC staining were performed on paraffin‐embedded sections using standard protocols. mIHC was performed using anti‐polycystin‐1 (sc‐130554, 1:50; Santa Cruz Biotechnology) and anti‐Daam1 antibodies (14876‐1‐AP, 1:8000; Proteintech).

### AFM‐Based Single‐Molecule Force Spectroscopy (AFM‐SMFS)

Silicon nitride AFM cantilevers (MLCT‐Bio‐DC, Bruker) were modified as previously described. Briefly, APTES‐treated tips and glass coverslips were functionalized with maleimide groups using sulfo‐SMCC (Thermo Fisher Scientific). ELP20 peptides (GL‐ELP20‐C or C‐ELP20‐NGL) were covalently conjugated using thiol‐maleimide chemistry. GPS‐tagged proteins (Fgβ‐(GB1)_2_‐GPS or CBM‐Sdrg) were immobilized using the OaAEP1(C247A) ligase. Force‐extension measurements were conducted using a Nanowizard4 AFM (JPK). Cantilever spring constants (≈40 pN·nm^−1^) were calibrated thermally. Unfolding events were recorded in PBS (pH 7.4) at room temperature, and the data were analyzed using JPK and Igor Pro 6.12 software. A wormlike chain model (persistence length = 0.4 nm) was used for curve fitting.

### In Vivo Tumor Models

Female BALB/c nude mice (5–8 weeks old) were subcutaneously injected with MDA‐MB‐231 cells with or without CAFs (5 × 10⁶ cells in 200 µL PBS). In the Daam1 inhibitor group, mice received daily subcutaneous injections of etoposide for seven consecutive days. After 4 weeks, the mice were sacrificed and tumor tissues were collected for IHC analysis. This study was approved by the Institutional Animal Care and Use Committee of Nanjing Medical University (No. IACUC‐2305039).

### Statistical Analysis

All statistical analyses were performed using GraphPad Prism 8.0. Data are presented as mean ± SEM. The correlation between Daam1 and polycystin‐1 expression was assessed using Pearson's correlation analysis (Figure 3l). One‐way analysis of variance was used to analyze RhoA activity, tumor weight, and collagen concentration (Figures 5a and 7h; Figure S1d, Supporting Information). AFM data were analyzed using the chi‐square test (Figure 6e; Figure S6c, Supporting Information). Clinicopathological data were analyzed using the chi‐square test or Fisher's exact test (Table 1). Unpaired Student's *t*‐test was used for comparisons that were not otherwise specified (Mann–Whitney U test if variances were unequal).

## Conflict of Interest

The authors declare no conflict of interest.

## Supporting information



Supporting Information

## Data Availability

The data that support the findings of this study are available from the corresponding author upon reasonable request.
