# Supplementary material for: Polycystin‐1 Mutant Alters Mechanotransduction in Response to Collagen and Extracellular Matrix Stiffness via Daam1‐Dependent Microfilament Remodeling
Source: Adv Sci (Weinh). 2025 Aug 11;12(41):e09846. doi: 10.1002/advs.202509846 (PMC12591123; doi:10.1002/advs.202509846)
Supplement: Supplementary file 1 — Supporting Information [file ADVS-12-e09846-s001.docx]

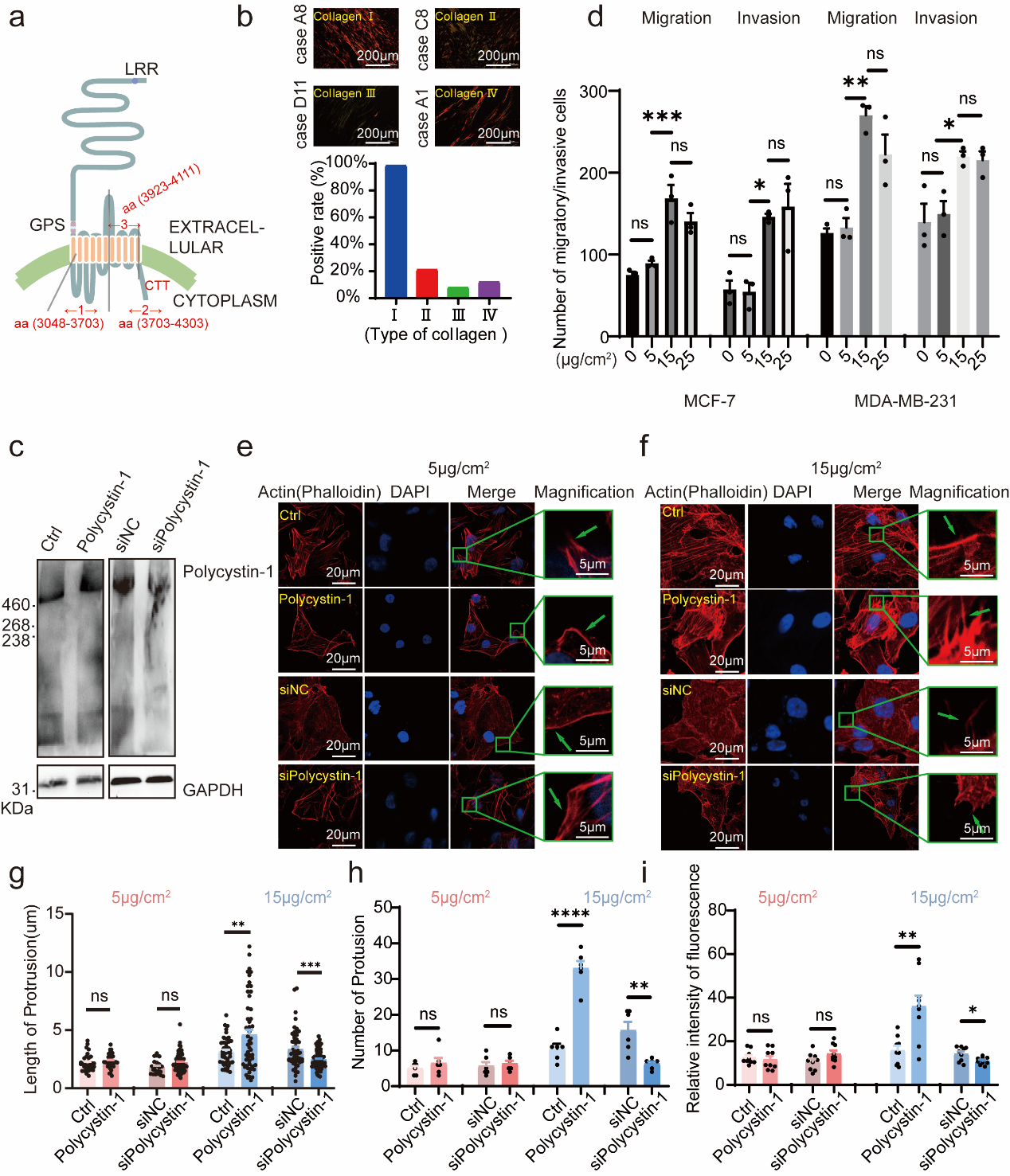


**Fig. S1 Polycystin-1 modulates collagen-induced microfilament assembly in tumor cells.**

**a** Polycystin-1 protein structure. LRR, leucine-rich repeat domain interacting with collagen; GPS, G protein-coupled receptor proteolytic site. The truncated constructs 1/2/3 correspond to HA-tagged fragments containing residues 3048–3703, 3703–4303, and 3923–4111, respectively. CTT, C-terminal tail.

**b** Collagen type I, II, III, and IV deposition in breast cancer pathological microarrays was detected by Sirius red staining and visualized under a polarized light microscope. Collagen I exhibited the highest positivity rate in tumor tissues. Magnification, 200×.

**c** Western blotting confirming overexpression and knockdown efficiency of polycystin-1 in MCF-7 cells transiently transfected with full-length polycystin-1 plasmids or siRNAs targeting polycystin-1.

**d** MCF-7 and MDA-MB-231 cells were seeded onto the upper chambers of Boyden inserts precoated with 0, 5, 15, or 25 μg/cm² collagen, or Matrigel for invasion assays, and allowed to migrate or invade for 24 h. n = 3. Cell migration and invasion differed significantly at 5 and 15 μg/cm² collagen (one-way ANOVA).

**e–i** Overexpression of polycystin-1 increased the length (**g**) and number (**h**) of cell protrusions, as well as intracellular microfilament fluorescence intensity (**i**), whereas knockdown decreased these parameters in response to 15 μg/cm² collagen. MDA-MB-231 cells were seeded on slides coated with 5 μg/cm² (**e**) or 15 μg/cm² collagen (**f**). F-actin and nuclei were stained with rhodamine-phalloidin (red) and DAPI (blue), respectively. Arrows indicate protrusions. Magnification, 630×. **P* < 0.05, ***P* < 0.01, ****P* < 0.001, *****P* < 0.0001; ns, not significant.


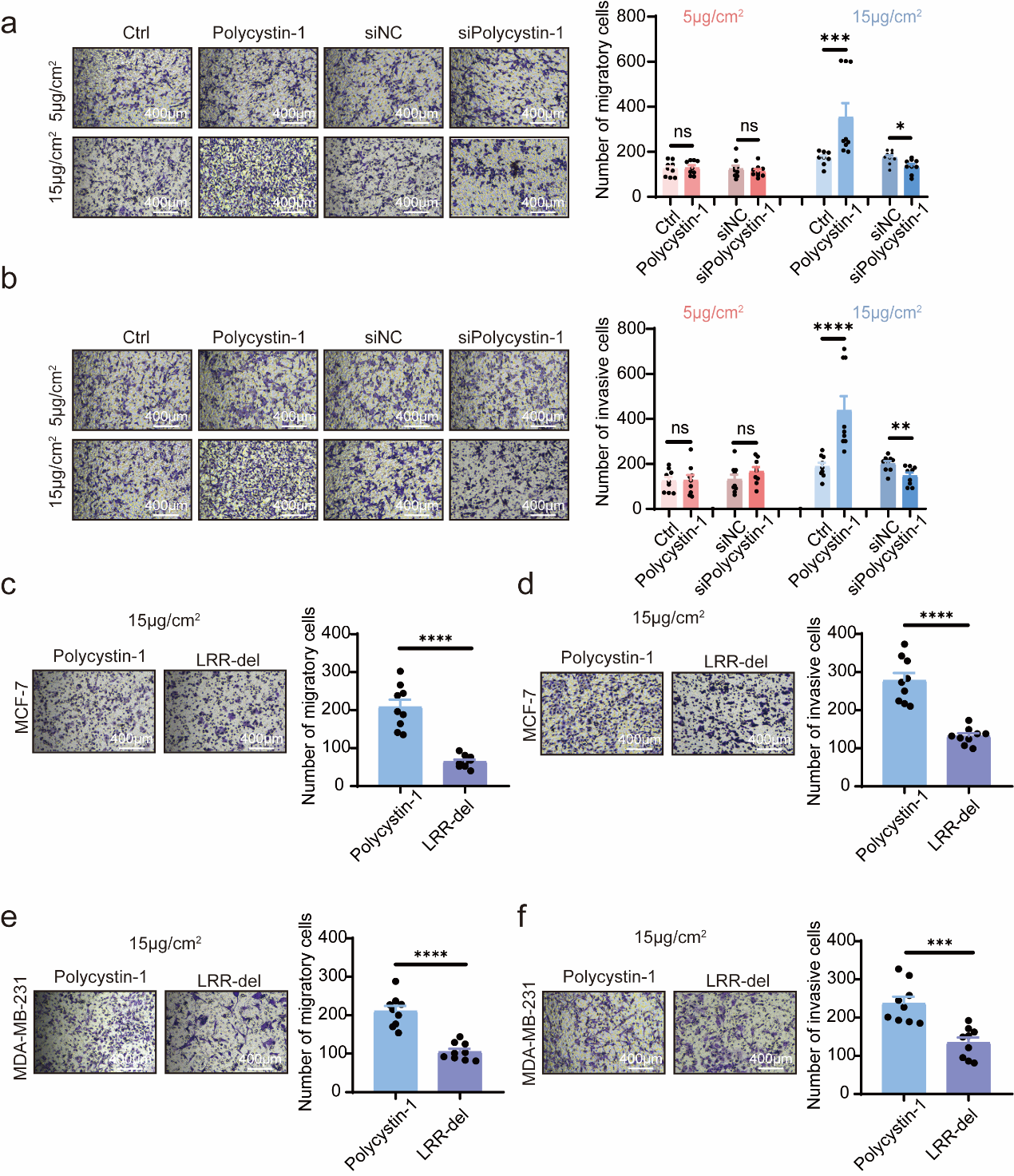


**Fig. S2 Collagen-induced tumor cell migration and invasion depend on the LRR domain of polycystin-1.**

**a, b** Overexpression of polycystin-1 promoted migration (**a**) and invasion (**b**), while polycystin-1 knockdown inhibited these processes in MDA-MB-231 cells in response to 15 μg/cm² collagen, but not at 5 μg/cm² collagen. Cells were seeded into Boyden chambers and allowed to migrate or invade for 24 h. n = 9. Magnification, 100×.

**c–f** Deletion of the LRR domain (LRR-del) in polycystin-1 abolished the 15 μg/cm² collagen-induced migration (**c, e**) and invasion (**d, f**) compared to full-length polycystin-1 overexpression. MCF-7 and MDA-MB-231 cells overexpressing LRR-del polycystin-1 or full-length polycystin-1 were subjected to Boyden chamber assay for 24 h (n = 9). Magnification, 100×. **P* < 0.05, ***P* < 0.01, ****P* < 0.001, *****P* < 0.0001; ns, not significant.


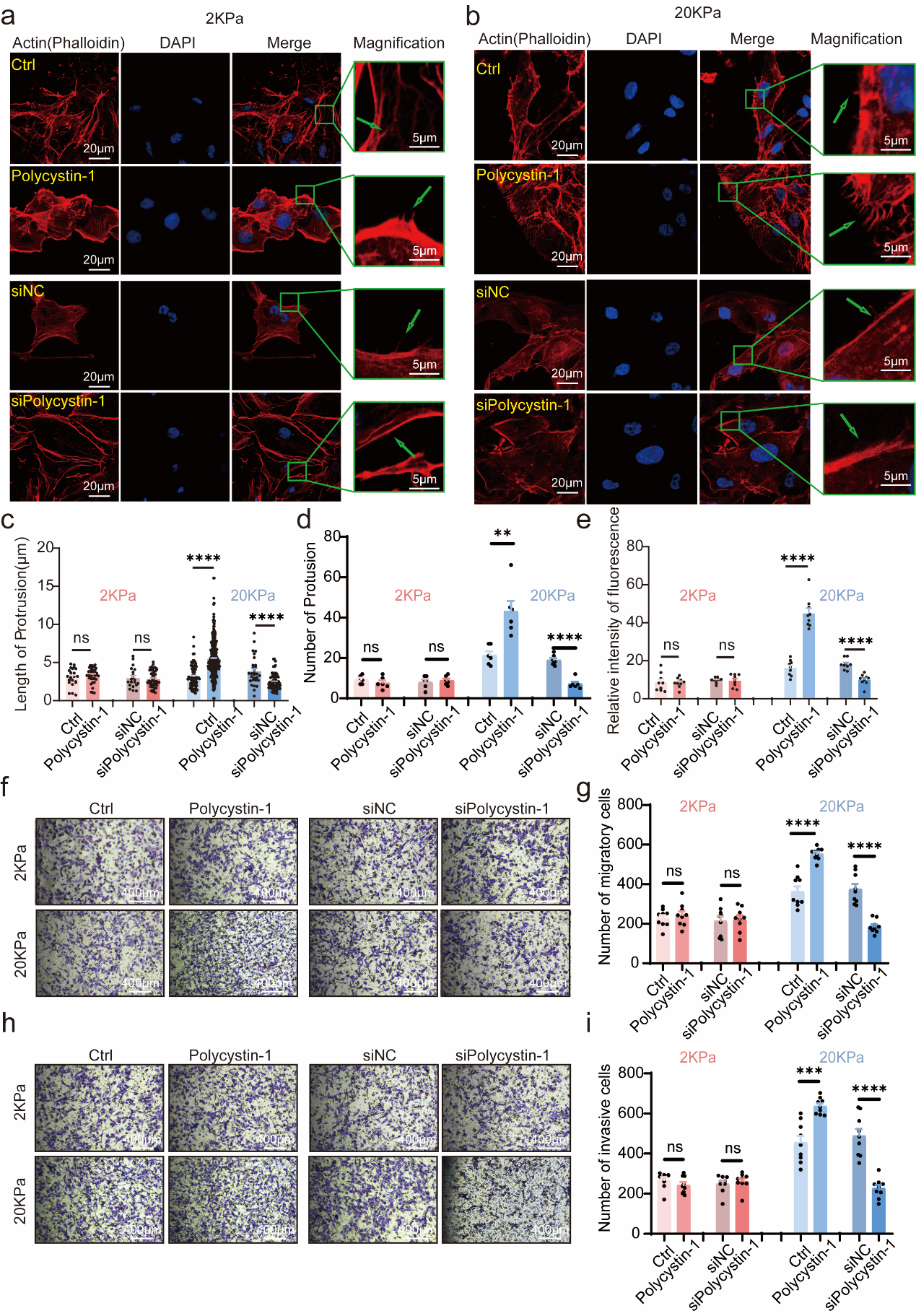


**Fig. S3 Polycystin-1 senses ECM stiffness and regulates microfilament remodeling and motility of MDA-MB-231 cells.**

**a–e** Overexpression of polycystin-1 enhances the length (**c**) and number (**d**) of cell protrusions and intracellular microfilament fluorescence intensity (**e**) in response to 20 kPa polyacrylamide gel (PAAG) but not 2 kPa PAAG. MDA-MB-231 cells were seeded on slides coated with 2 kPa (**a**) or 20 kPa PAAG (**b**). F-actin and nuclei were stained with rhodamine-phalloidin (red) and DAPI (blue), respectively. Arrows indicate protrusions. Magnification, 630×.

**f–i** Overexpression of polycystin-1 promoted cell migration (**f, g**) and invasion (**h, i**), while knockdown inhibited migration and invasion in response to 20 kPa PAAG but not 2 kPa PAAG. Cells were seeded onto Boyden chambers following 24 h of treatment with 2 or 20 kPa PAAG and then allowed to migrate or invade for 24 h. n = 9. Magnification, 100×. ***P* < 0.01, ****P* < 0.001, ***P* < 0.0001; ns, not significant.


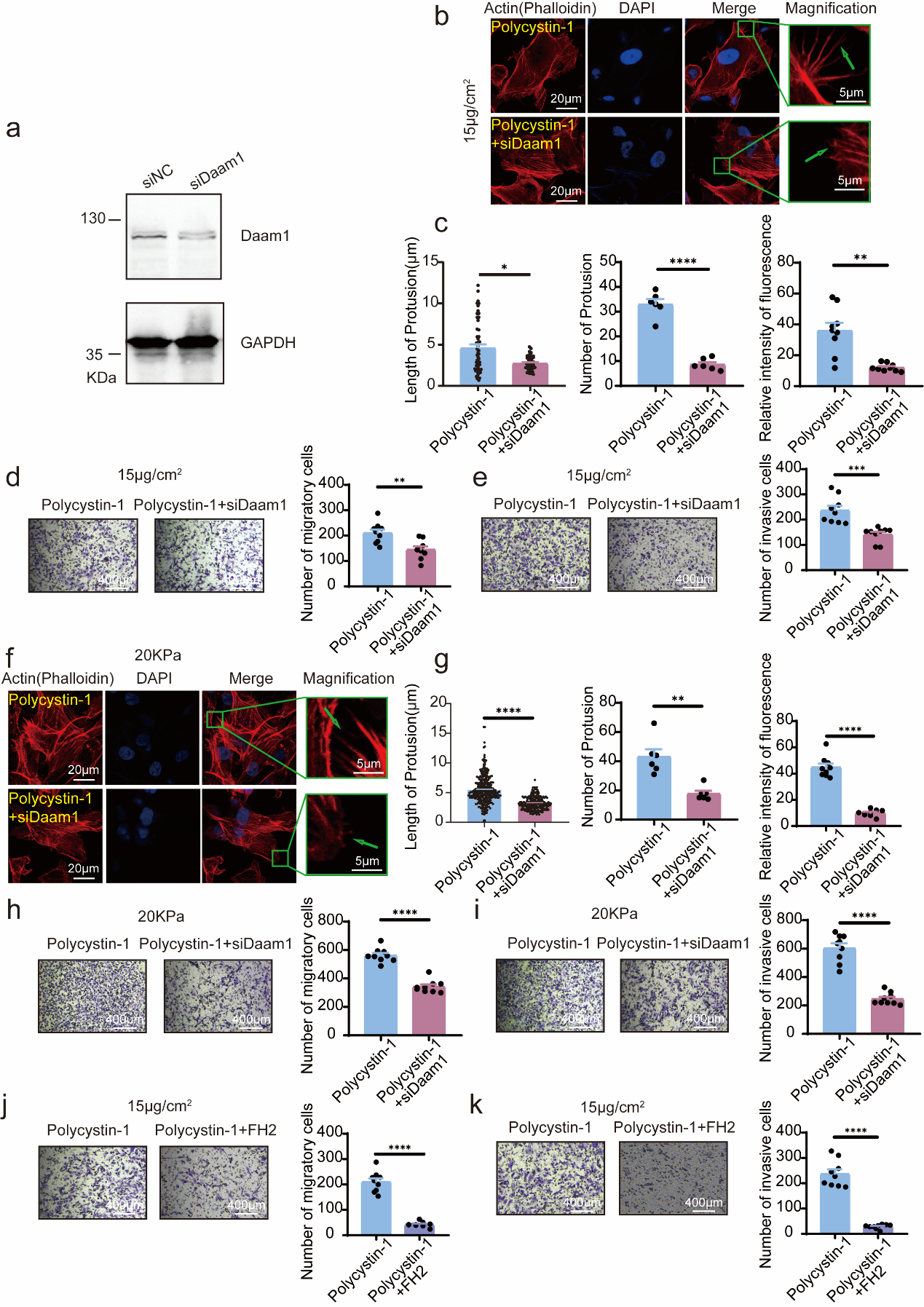


**Fig. S4 Polycystin-1 interacts with Daam1 to remodel microfilaments and regulate motility of MDA-MB-231 cells.**

**a** Verification of Daam1 knockdown efficiency by western blotting in MCF-7 cells transiently transfected with Daam1-targeting siRNAs.

**b, c** Overexpression of polycystin-1 combined with Daam1 knockdown decreased the length and number of protrusions as well as the relative fluorescence intensity of intracellular microfilaments in response to 15 μg/cm² collagen. MDA-MB-231 cells were seeded on slides coated with 15 μg/cm² collagen (**b**). F-actin and nuclei were stained with rhodamine-phalloidin (red) and DAPI (blue), respectively. The length and number of cell protrusions and relative fluorescence intensity of the intracellular microfilaments were quantified using laser scanning confocal microscopy (**c**). Arrows indicate protrusions. Magnification, 630×.

**d, e** Overexpression of polycystin-1 combined with Daam1 knockdown inhibited collagen-induced cell migration (**d**) and invasion (**e**) compared with polycystin-1 overexpression alone. MDA-MB-231 cells with indicated treatments were seeded into Boyden chambers coated with 15 μg/cm² collagen and allowed to migrate or invade for 24 h. n=9. Magnification, 100×.

**f, g** Similar effects were observed in cells seeded on 20 KPa PAAG-coated slides: combined polycystin-1 overexpression and Daam1 knockdown decreased protrusion length, number, and microfilament fluorescence intensity (**g**) compared with polycystin-1 overexpression alone. F-actin and nuclear staining were performed as described previously (**f**). Magnification, 630×.

**h, i** Combined polycystin-1 overexpression and Daam1 knockdown suppressed 20 KPa PAAG-induced cell migration (**h**) and invasion (**i**) relative to polycystin-1 overexpression alone. Cells were treated with 20 KPa PAAG for 24 h prior to the Boyden chamber assays. n=9. Magnification, 100×.

**j, k** Co-expression of polycystin-1 and the FH2 domain of Daam1 inhibited 15 μg/cm² collagen-induced cell migration (**j**) and invasion (**k**) compared to polycystin-1 overexpression alone. The cells were seeded in Boyden chambers and incubated for 24 h (n=9). Magnification, 100×. **P* < 0.05, ***P* < 0.01, ****P* < 0.001, *****P* < 0.0001.


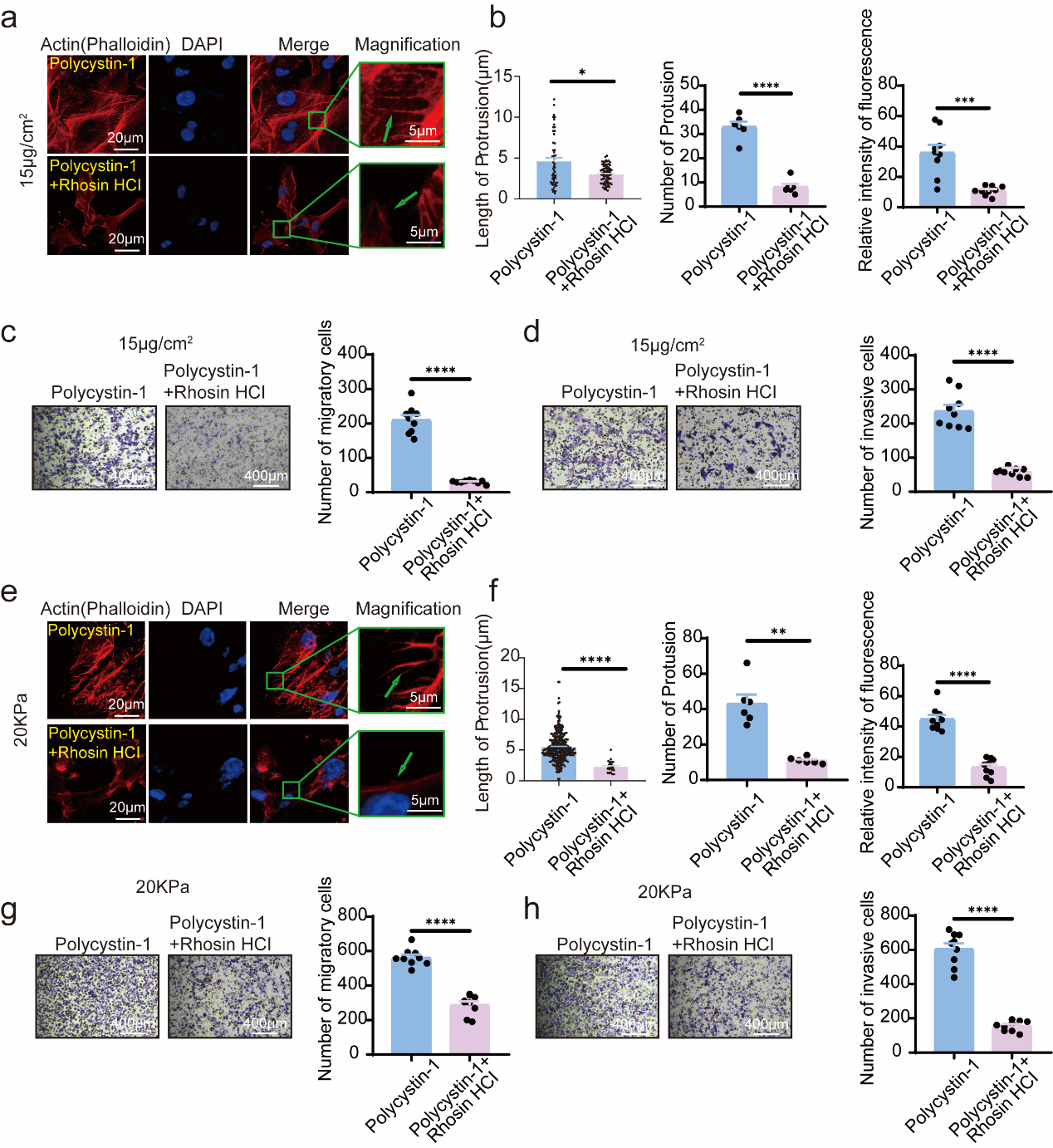


**Fig. S5 Polycystin-1/Daam1/RhoA axis regulates microfilament remodeling and motility of MDA-MB-231 cells.**

**a, b** Treatment with rhosin HCl, a RhoA inhibitor, decreased the length and number of protrusions and relative fluorescence intensity (**b**) of intracellular microfilaments in polycystin-1 overexpressing MDA-MB-231 cells seeded on 15 μg/cm² collagen-coated slides (**a**). The cells were then stained with Rhodamine-phalloidin and DAPI. Arrows indicate protrusions. Magnification, 630×.

**c, d** Rhosin HCl significantly inhibited polycystin-1-induced migration (**c**) and invasion (**d**) in Boyden chamber assays with collagen coating. Cells were treated with or without 30 μM rhosin HCl during 24 h incubation. n=9. Magnification, 100×.

**e, f** On 20 KPa PAAG-coated slides (**e**), rhosin HCl similarly reduced the protrusion length, number, and microfilament fluorescence intensity (**f**) in polycystin-1-overexpressing cells. Magnification, 630×.

**g, h** Rhosin HCl inhibited polycystin-1-driven cell migration (**g**) and invasion (**h**) on 20 KPa PAAG in Boyden chambers. Cells were treated as described above. n=9. Magnification, 100×. **P* < 0.05, ***P* < 0.01, ****P* < 0.001, *****P* < 0.0001.


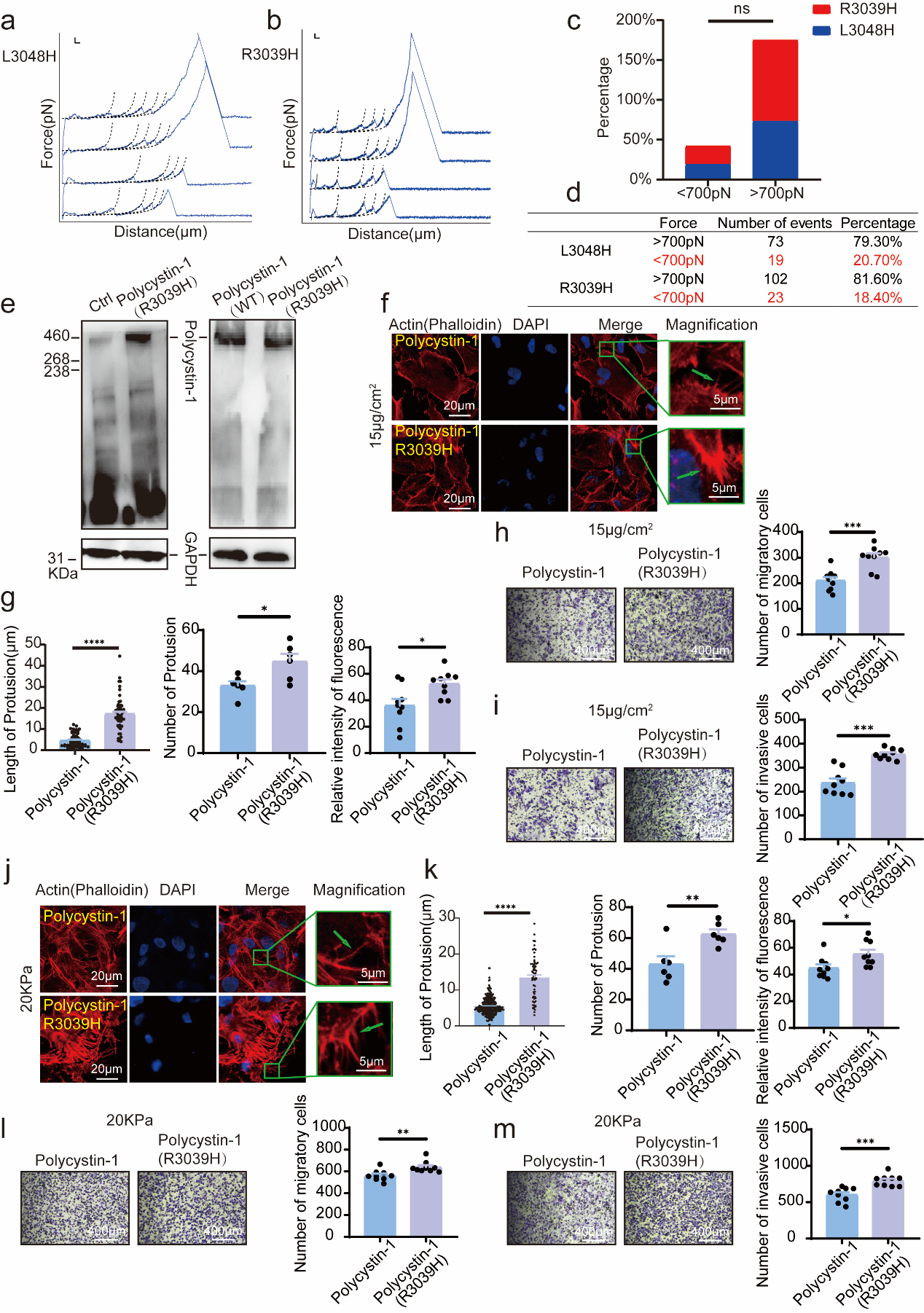


**Fig. S6 AFM-SMFS analysis of polycystin-1 GPS domain mutants and their effects on microfilament remodeling and motility of MDA-MB-231 cells.**

**a, b** Single-molecule force spectroscopy (SMFS) analysis of GPS domain mutants of polycystin-1 L3048H (**a**) and R3039H (**b**) using atomic force microscopy (AFM).

**c, d** Quantification of cleavage events in polycystin-1 L3048H and R3039H mutants. Statistical analyses were performed using the chi-square test.

**e** Western blot verification of the overexpression efficiency of polycystin-1 WT and R3039H mutants in MCF-7 cells.

**f, g** Compared to WT, overexpression of polycystin-1 R3039H increased the length and number of protrusions as well as relative fluorescence intensity (**g**) of intracellular microfilaments in MDA-MB-231 cells seeded on 15 μg/cm² collagen-coated slides (**f**). F-actin and nuclei were stained, as previously described. Arrows indicate protrusions. Magnification, 630×.

**h, i** Polycystin-1 R3039H enhanced collagen-induced migration (**h**) and invasion (**i**) compared to the WT in Boyden chamber assays. n=9. Magnification, 100×.

**j, k** On 20 KPa PAAG-coated slides (**j**), polycystin-1 R3039H overexpression increased the protrusion length, number, and microfilament fluorescence intensity (**k**). Magnification, 630×.

**l, m** Polycystin-1 R3039H promoted 20 KPa PAAG-induced migration (**l**) and invasion (**m**) compared to the WT. The cells were pretreated with 20 KPa PAAG for 24 h before the Boyden assay. n=9. Magnification, 100×. **P* < 0.05, ***P* < 0.01, ****P* < 0.001, *****P* < 0.0001. ns, no significant difference.


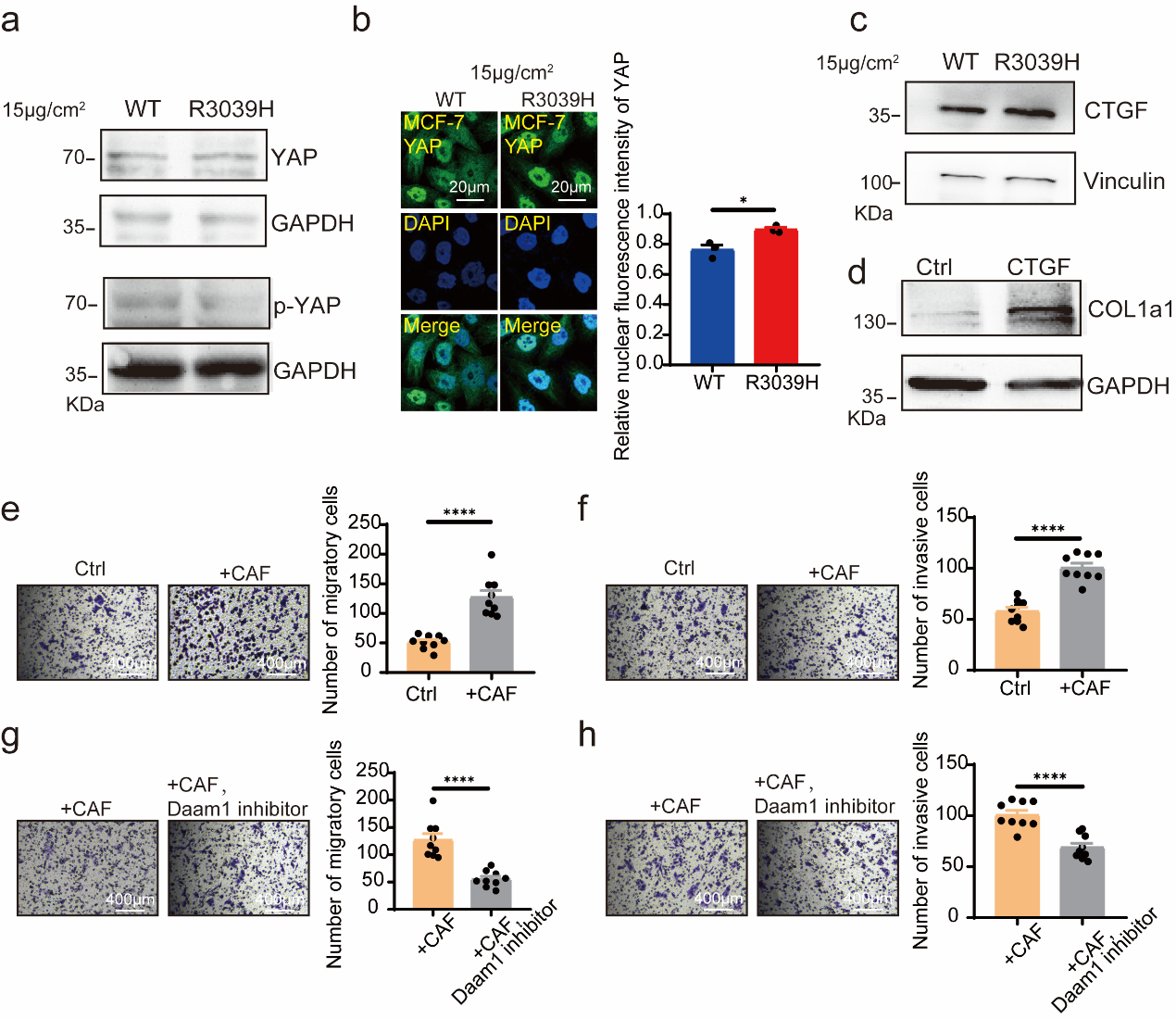


**Fig. S7 Polycystin-1 R3039H enhances CTGF expression by promoting nuclear translocation of YAP in MCF-7 cells.**

**a** Western blot analysis showing decreased p-YAP (S127) levels in MCF-7 cells overexpressing polycystin-1 R3039H compared to WT in response to 15 μg/cm² collagen. Whole-cell lysates were probed with anti-YAP1 and anti-p-YAP (S127) antibodies. The results shown are representative of three independent experiments.

**b** Immunofluorescence staining showing increased nuclear localization of YAP in polycystin-1 R3039H-overexpressing MCF-7 cells relative to WT after 15 μg/cm² collagen treatment. Anti-YAP1 antibody was used for staining. Magnification, 630×.

**c** Western blot demonstrating elevated CTGF expression in polycystin-1 R3039H-overexpressing MCF-7 cells compared to WT under 15 μg/cm² collagen stimulation. The results shown are representative of three independent experiments.

**d** Western blot showing increased collagen I (COL1a1) expression in cancer-associated fibroblasts (CAFs) treated with 1.695 μg/ml CTGF for 24 h. The results shown are representative of three independent experiments.

**e, f** Co-culture of MCF-7 cells with CAFs significantly enhanced tumor cell migration (**e**) and invasion (**f**) in Boyden chambers. MCF-7 cells were seeded in the upper chambers and CAFs in the lower chambers, and migration and invasion assays were conducted for 24 h. n=9. Magnification, 100×.

**g, h** Treatment with the Daam1 inhibitor etoposide (270 μM) significantly reduced migration (**g**) and invasion (**h**) of tumor cells in MCF-7/CAF co-culture assays. n=9. Magnification, 100×. *****P* < 0.0001.

**Table S1. GPS domain mutant sites in the Uniprot database**

| Genotype | Mutant sites | Type of mutation |
| --- | --- | --- |
| c.9090G>T | p.L3030= | Substitution - coding silent |
| c.9124G>A | p.R3039H | Substitution - missense |
| c.9153C>T | p.F3051= | Substitution - coding silent |

**Table S2. Research Resource Identifiers (RRIDs) of cell lines and reagents used in this study.**

| Reagent or Resource | Source/Catalog Number | RRID |
| --- | --- | --- |
| MCF-7 | the National Collection of Authenticated Cell Cultures (Shanghai, China) | CVCL_0031 |
| MDA-MB-231 |  | CVCL_0062 |
| HEK293T |  | CVCL_0063 |
|  |  |  |
| pCI-PC1-Flag plasmid | Addgene, #21369 | Addgene_21369 |
|  |  |  |
| Anti-Polycystin-1 | Santa Cruz, sc-130554 | AB_2163355 |
| Anti-Daam1 | Proteintech, 14876-1-AP | AB_2089444 |
| Anti-GAPDH | Proteintech, 10494-1-AP | AB_2263076 |
| Anti-FLAG | Proteintech, 20543-1-AP | AB_11232216 |
| Anti-HA | Proteintech, 51064-2-AP | AB_11042321 |
| Anti-phospho-YAP (S127) | Abclonal, AP1436 | N/A |
| Anti-YAP | Proteintech, 13584-1-AP | AB_2218915 |
| Anti-CTGF | Santa Cruz, sc-565970 | N/A |
| Anti-COL1a1 | Proteintech, 67288-1-Ig | AB_2882554 |
